# Combined Physiological and Transcriptomic Analyses of the Effects of Exogenous Trehalose on Salt Tolerance in Maize (*Zea mays* L.)

**DOI:** 10.3390/plants13243506

**Published:** 2024-12-16

**Authors:** Jingyi He, Hongliang Tang

**Affiliations:** School of Life Sciences, Hebei University, Baoding 071002, China; xxxxxelie@163.com

**Keywords:** maize (*Zea mays* L.), salt stress, trehalose, antioxidant enzymes, photosynthesis, sugar metabolism, abscisic acid

## Abstract

Soil salinization severely affects the quality and yield of maize. As a C4 plant with high efficiency in utilizing light and carbon dioxide, maize (*Zea mays* L.) is one of the most important crops worldwide. This study aims to investigate the pathways and mechanisms by which trehalose mediates the improvement of salt tolerance in maize through a combined analysis of physiology and transcriptomics. The results indicate that foliar application of trehalose treatment significantly increased maize biomass and antioxidant enzyme activity while reducing the H_2_O_2_ and Na^+^/K^+^ ratios in both the aerial and underground parts of the plant. Additionally, trehalose enhanced the total secretion of organic acids from maize roots, improving the soil microenvironment for maize growth under salt stress and alleviating Na^+^ toxicity. Transcriptomic data revealed that under salt stress, most differentially expressed genes (DEGs) were enriched in pathways related to photosynthesis, abscisic acid signaling, and sugar metabolism, and trehalose application increased the expression levels of these pathways, thereby mitigating the growth inhibition caused by salinity. This study elucidates mechanisms for enhancing salt tolerance in maize, providing theoretical support for improving its resilience and offering innovative strategies for utilizing a wide range of saline-alkali land.

## 1. Introduction

Maize (*Zea mays* L.) is the world’s leading grain crop [1] and a major source of animal feed. Due to limitations in climate and arable land [2], enhancing salt tolerance in maize has become a pressing issue in agricultural production. Therefore, alleviating stress in maize and improving both its yield and nutritional quality are of great significance. In addition to developing salt-tolerant maize varieties [3,4], nutritional interventions offer a key approach to improving maize’s resistance to salinity and enhancing the utilization of saline soils [5,6,7]. Studies have shown that the application of exogenous substances such as melatonin, salicylic acid, sucrose, and polyamines is an important strategy for increasing plant stress resistance [8,9,10].

Soil is one of the most complex and diverse ecosystems on Earth [11]. In addition to its ecological service values such as carbon storage, greenhouse gas regulation, and flood prevention, the soil ecosystem provides 98.8% of human food [12]. However, with the increasing impact of global warming, soil salinization events caused by both natural and human factors are becoming more frequent. Soil salinization, which adversely affects plant physiology and soil physical and chemical properties, poses a global threat to agriculture and the environment [13]. The presence of large amounts of soluble salts in soil is referred to as soil salinization [14]. Naturally, saline soils are often found in arid and semi-arid regions, where low precipitation and high evaporation rates, combined with topographic and climatic factors, cause deep soil salts to rise to the surface through capillary water movement [15]. These salts accumulate on the surface, as they cannot evaporate with transpiration, eventually leading to soil salinization. Additionally, human factors such as improper irrigation, poor drainage, and excessive fertilization exacerbate the severity of soil salinization [16].

Salt stress is a major factor that limits plant growth and photosynthesis [17]. Plants absorb large amounts of water from the soil through their roots, driven by a water potential gradient, where water moves from areas of higher water potential to areas of lower water potential, participating in numerous critical physiological processes [18]. The adverse effects of soil salinization on plants are multifaceted, including inhibiting plant growth and development, disrupting physiological metabolic processes, and causing DNA damage. Salt stress initially induces the accumulation of abscisic acid (ABA) and the closure of stomata, which subsequently leads to the excessive production of reactive oxygen species (ROS) [19]. This overproduction can cause oxidative damage to cell membranes, the degradation of chlorophyll, a reduction in photosynthetic performance, the obstruction of metabolite transport, and an imbalance in physiological processes. Ultimately, cell expansion is restricted, leading to stunted growth or even a complete halt in growth [13].

Trehalose is a non-reducing disaccharide formed by the linkage of two glucose residues through an α-α-(1→1) bond [20]. It was first discovered in rye infected with ergot in 1832. Since then, it has been detected in a wide range of organisms, including bacteria, fungi, invertebrates, and plants [21], often associated with stress resistance phenotypes [22]. In plants, the biosynthesis of trehalose involves a two-step pathway that includes TPS and TPP enzymes, while TRE degrades trehalose into two glucose molecules [23]. The trehalose biosynthesis intermediate, Tre6P, regulates the allocation of photosynthates between sucrose and organic and amino acids by controlling the post-translational activation of phosphoenolpyruvate carboxylase (PEPC) and nitrate reductase (NR) [24]. In heterotrophic tissues, Tre6P inhibits SnRK1 activity through interactions with SnAK kinase and unknown protein factors [25]. The TPP promoter contains sequences involved in ABA signaling [26], as well as genes related to hormones, such as auxins, gibberellins (GA), salicylic acid (SA), and methyl jasmonate (MJ), and stress responses [27,28]. Compared with other disaccharides, trehalose possesses unique physical properties, as both of its reducing moieties are involved in glycosidic bond formation [29,30]. Therefore, there are different hypotheses regarding the direct actions of trehalose on membrane and macromolecule protection: water replacement, glass formation, and chemical stability [31]. According to water replacement theory, all biomacromolecules are typically stabilized by the formation of hydrogen bonds around the macromolecules. It has been reported that trehalose and its derivatives are important regulators of gene expression in plants, with trehalose treatment altering the expression of stress-related genes [32]. Although higher plants do not accumulate large amounts of trehalose, the gene family associated with trehalose synthesis is still highly expressed, indicating that trehalose and its metabolites play important roles in plants [33].

Transcriptomics refers to the study of gene transcription within cells at a holistic level, including research on the functions of non-coding regions, transcription structures, gene transcription levels, and the identification of new transcription regions [34]. In multidisciplinary projects, transcriptomics is often the first omics technology to be applied. It can generate information about which genes are expressed at what levels, as well as provide insights into the different transcript isoforms utilized. Preliminary analyses conducted through microarray or RNA sequencing (RNA-seq) can indicate the suitability or practicality of other omics techniques, such as proteomics, glycomics, and metabolomics [35]. However, transcriptomics is highly complex and dynamic, with changes influenced by various factors [36]. Analyzing the impact of trehalose on various biological processes and metabolic pathways in maize at the gene expression level can help us gain a deeper understanding of the transcriptional regulatory networks associated with trehalose in plants.

Trehalose and its biosynthesis pathway play significant roles and regulatory functions in plant responses to stress. Both trehalose and its biosynthetic pathway enhance plant stress tolerance, an effect often attributed to the unique chemical properties of trehalose and the upstream signaling involved in its synthesis. However, the comprehensive pathways through which trehalose itself and its downstream signaling in enhancing plant salt tolerance remain largely unexplored, and studies on the related pathways and mechanisms are scarce. This study focuses on maize and its response to trehalose, utilizing both hydroponic and soil cultivation methods. It investigates the effects of trehalose on the growth parameters, reactive oxygen accumulation, osmotic regulators, hormonal regulation, enzyme activities, and gene expression patterns of maize under salt stress. Additionally, it preliminarily explores the influence of signaling molecules in the trehalose metabolic pathway on hormone synthesis, based on transcriptome sequencing results and Quantitative Real-Time PCR (qRT-PCR) data, aiming to elucidate the regulatory mechanisms of trehalose in maize’s stress response under salt conditions.

## 2. Results

### 2.1. Growth Indicators

**Figure 1:**
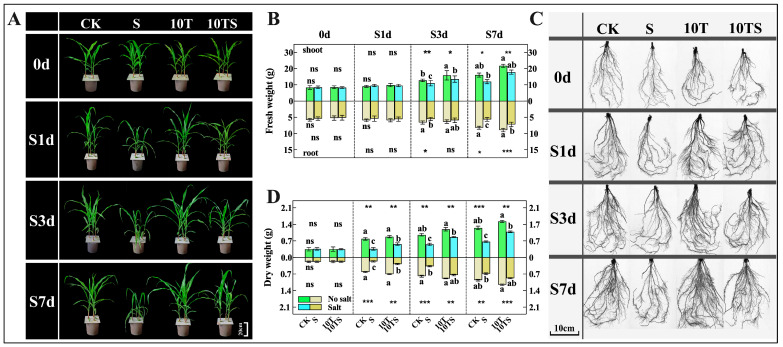

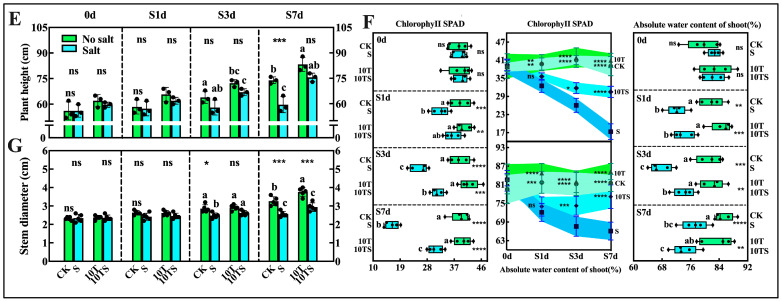
Growth parameters of maize plants at different sampling time points in the hydroponic experiment under salt stress and trehalose treatment. Photos were taken, and samples were collected on the 15th (0d), 19th (S1d), 21st (S3d), and 25th (S7d), days of maize growth to measure various growth parameters: maize seedling phenotype (**A**), fresh weight (**B**), root system phenotype (**C**), dry weight (**D**), plant height (**E**), chlorophyll content, relative water content, and daily dynamic changes in the aerial parts (**F**), and stem diameter (**G**). Note: date showing the means ± standard deviation (SD). ns: no significant difference. Asterisks: using the *t*-test, indicating the significance levels of differences between CK (the control) and S (the salt stress), and 10T (the application of trehalose under normal nutritional) and 10TS (the application of trehalose under salt stress): *: a highly significant difference at * *p* < 0.05, ** at *p* < 0.01, *** at *p* < 0.001, and **** at *p* < 0.0001. Lowercase letters: using the one-way ANOVA (Turkey, Duncan), indicating significant differences (*p* < 0.05) between groups under salt stress and trehalose treatment (*n* = 3).

Plant morphology is significant in assessing salt stress, as salt stress significantly inhibits the growth and biomass accumulation of maize (Figure 1). Compared with the control (CK), maize subjected to 100 mM NaCl salt stress (S) showed significant reductions in fresh weight (Figure 1B), dry weight (Figure 1D), plant height (Figure 1E), stem diameter (Figure 1G), relative chlorophyll content (Figure 1F), and relative water content (Figure 1F). These negative effects were particularly pronounced on the seventh day of stress (S7d), with reductions of −33.65, −27.53, −19.37, −21.47, −57.19, and −19%, respectively, severely impacting the growth phenotype of the maize. However, the 10TS treatment significantly rescued the reductions in the above growth parameters caused by salt stress, with recovery trends reaching 14.84 to 82.42%. Additionally, the application of trehalose under normal nutritional conditions also promoted maize growth, with increases in various indicators ranging from 4.53 to 20.29% compared with the control. This indicates that trehalose is beneficial for maize growth to some extent.

High salt concentrations initially disrupt the osmotic balance between the cytoplasm and the growth matrix, leading to physiological drought and inhibiting root growth (Table S1). In this study, salt stress had a highly significant detrimental effect on all root parameters examined, except for root length, with these effects being evident from the first day of salt stress (S1d) and reaching the most significant differences by S7d. In a hydroponic environment with 100 mM NaCl (S) at S7d, root length, average root diameter, root surface area, and root volume were reduced by −36.74, −10, −22.82, and −24.14%, respectively, compared with the control. However, the application of 10 mM trehalose alone significantly altered these root parameters, indicating that exogenous trehalose under normal nutritional conditions benefits maize root growth, with increases of 32.55, 10, 30.05, and 31.53% in root length, average root diameter, root surface area, and root volume, respectively (S7d). A similar effect was observed under salt stress conditions, where the application of trehalose alleviated the inhibitory effects of salt stress on maize root growth, with the aforementioned parameters increasing by 29.53, 8.3, 20.07, and 24.68% (S7d), demonstrating a significant positive impact on maize root growth.

### 2.2. Osmotic System

**Figure 2:**
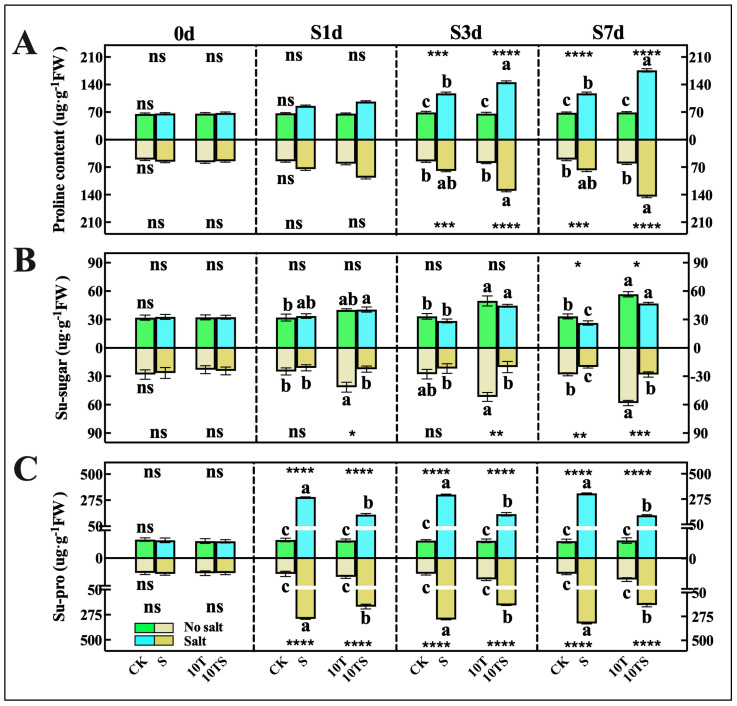
Contents of osmotic substances in maize at different sampling time points in the hydroponic experiment under salt stress and trehalose treatment. The levels of proline (**A**), soluble sugar (**B**), and soluble protein (**C**) were measured at each time point. Note: date showing the means ± standard deviation (SD). ns: no significant difference. Asterisks: using the *t*-test, indicating the significance levels of differences between CK (the control) and S (the salt stress), or 10T (the application of trehalose under normal nutritional) and 10TS (the application of trehalose under salt stress): *: a highly significant difference at * *p* < 0.05, ** at *p* < 0.01, *** at *p* < 0.001, and **** at *p* < 0.0001. Lowercase letters: using the one-way ANOVA (Turkey, Duncan), indicating significant differences (*p* < 0.05) between groups under salt stress and trehalose treatment (*n* = 3).

The roots are directly exposed to salt stress, so the changes in osmotic substances and the degree of membrane permeability damage are more pronounced than those in the aerial parts of the plant. On the 7th day of salt stress (S7d), the differences in the maize osmotic regulation system were significantly more pronounced than at the first three sampling times (Figure 2). The extent of electrolyte leakage in the underground parts of the maize was more severe than in the aerial parts.

Under salt stress, compared with CK, the proline levels (Figure 2A) in the aerial and root parts of the S group significantly increased, with increases of 72.49 and 53.97%, respectively, by S7d. The accumulation of proline in the 10TS group was lower than that in the S group but still significantly higher than that in the CK group.

The soluble sugar (Figure 2B) content in the plants was largely influenced by the application of exogenous trehalose, with both the 10T and 10TS treatments showing significantly higher levels than the group without trehalose. The soluble sugar content in the 10T group increased by approximately 70% compared with CK (S7d). However, the data showed that the soluble sugar content in the 10TS group was significantly lower than that in the 10T group, being only about 40% higher than CK, which may be related to energy deficits in maize growth under salt stress. The soluble protein (Figure 2C) content in the maize under salt stress significantly increased starting from S1d, with soluble proteins in the aerial and underground parts increasing by 9 to 11 times by S7d compared with the control. While the soluble protein content in the maize under trehalose application also increased, it was only 3 to 4 times higher.

### 2.3. Antioxidant System

**Figure 3:**
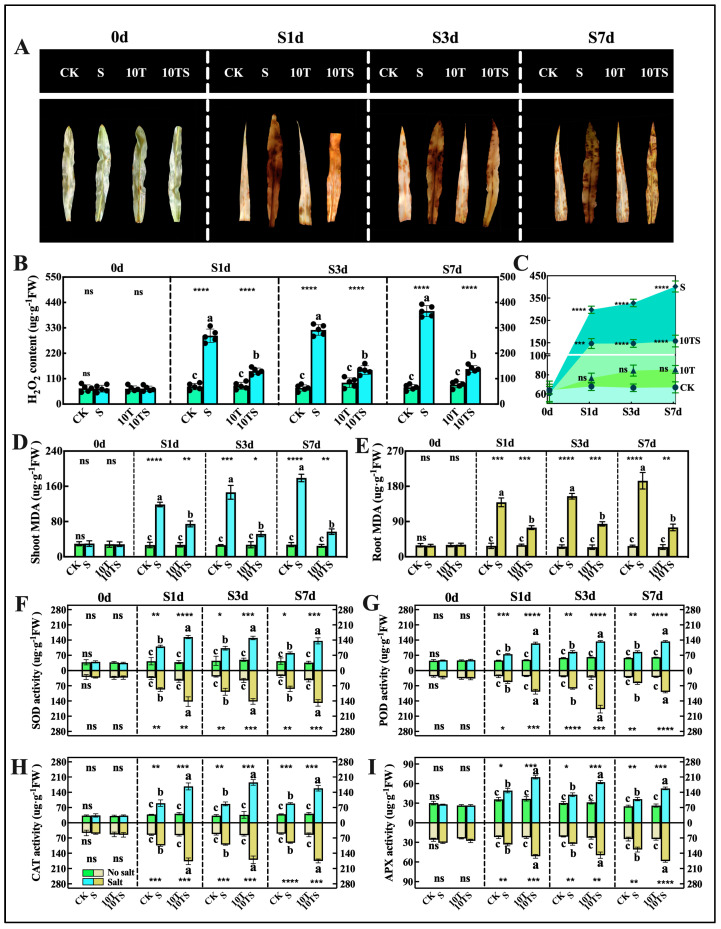
The hydrogen peroxide staining phenotype of maize leaves (**A**), hydrogen peroxide content (**B**), daily variation of hydrogen peroxide content (**C**), shoot MDA (**D**), root MDA (**E**), SOD activity (**F**), POD activity (**G**), CAT activity (**H**), and APX activity (**I**) were measured. Note: date showing the means ± standard deviation (SD). ns: no significant difference. Asterisks: using the *t*-test, indicating the significance levels of differences between CK (the control) and S (the salt stress), or 10T (the application of trehalose under normal nutritional) and 10TS (the application of trehalose under salt stress): *: a highly significant difference at * *p* < 0.05, ** at *p* < 0.01, *** at *p* < 0.001, and **** at *p* < 0.0001. Lowercase letters: significant differences (*p* < 0.05, one-way ANOVA) between groups under salt stress and trehalose treatment (*n* = 3).

The extent of electrolyte leakage in the underground parts of the maize was more severe than in the aerial parts. Salt stress caused oxidative damage in the plants, significantly increasing the H_2_O_2_ content (Figure 3A–C) in the leaves to five times that of the control group. The application of trehalose mitigated this damage, with the H_2_O_2_ content in the 10TS group being 1.3 times that in the CK group (S7d). Under salt stress, the malondialdehyde (MDA) content (Figure 3D–E) in the shoots and roots of the maize significantly increased (S7d), reaching 5.48 times and 6.1 times that of the control group, respectively, by S7d. However, after trehalose supplementation, the accumulation of MDA significantly reduced, decreasing by approximately 1.5 to 2 times (S7d). To counteract the negative effects of reactive oxygen species, plants produce antioxidant enzymes. In this experiment, salt stress significantly increased the Superoxide dismutase (SOD), Peroxidase (POD), Catalase (CAT), and Ascorbate Peroxidase (APX) activities in the maize leaves and roots by 40% to 120% (Figure 3F–I) compared with unstressed plants (S7d). Following the application of exogenous trehalose, enzyme activities were further enhanced, increasing by 43% to 79% compared with the salt-stressed conditions (Figure 3F–I).

### 2.4. K^+^ and Na^+^ Homeostasis

**Figure 4:**
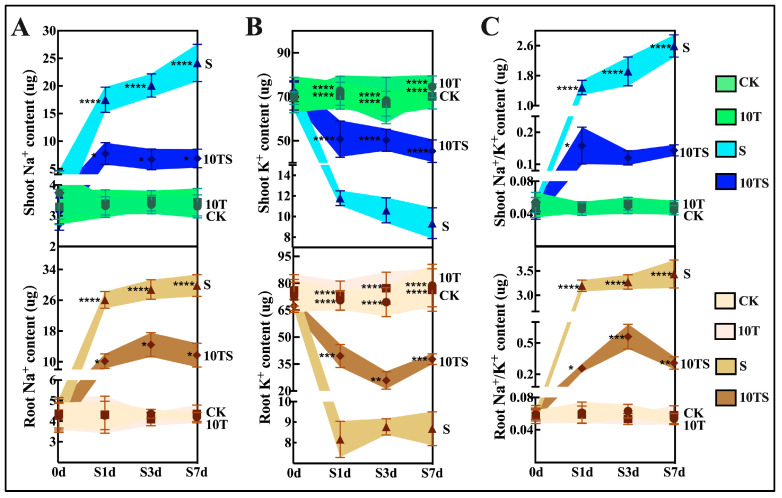
Transport of K^+^ and Na^+^ in maize at different sampling time points in the hydroponic experiment under salt stress and trehalose treatment. The Na^+^ content (**A**), K^+^ content (**B**), and the changes in Na^+^/K^+^ ratio (**C**) were measured. Note: date showing the means ± standard deviation (SD). ns: no significant difference. Asterisks: using the *t*-test, indicating the significance levels of differences between CK (the control) and S (the salt stress), or 10T (the application of trehalose under normal nutritional) and 10TS (the application of trehalose under salt stress): *: a highly significant difference at * *p* < 0.05, ** at *p* < 0.01, *** at *p* < 0.001, and **** at *p* < 0.0001. Lowercase letters: using the one-way ANOVA (Turkey, Duncan), indicating significant differences (*p* < 0.05) between groups under salt stress and trehalose treatment (*n* = 3).

Salt stress primarily affects the ion transport in roots by causing an excessive accumulation of sodium ions, leading to an imbalance in the dynamic equilibrium of K^+^ and Na^+^. Under salt stress, the Na^+^ content in the roots and leaves gradually increased with time (Figure 4), showing significant differences from the control group starting from S1d, reaching six times the Na^+^ content in the CK group at S7d. In the 10TS group, Na^+^ concentrations significantly decreased, with reductions of 1.54 times in the leaves and 2.98 times in the roots (Figure 4A). Salt stress severely inhibited K^+^ absorption in the maize (S7d), with the K^+^ content in the leaves and roots decreasing by 87.49 and 88.99% (Figure 4B), respectively. However, the application of trehalose significantly alleviated this stress response. The effects of salt stress on the K^+^ and Na^+^ content directly impacted the Na^+^/K^+^ ratio in the plants, which decreased by about 98% (S7d) compared with the control group (Figure 4C). After the application of exogenous trehalose, this suppression was largely mitigated, with the 10TS group showing only a 70% reduction compared with the CK group (S7d).

### 2.5. Physiological Characteristics of Maize Seedlings in Soil Culture Experiments

**Figure 5:**
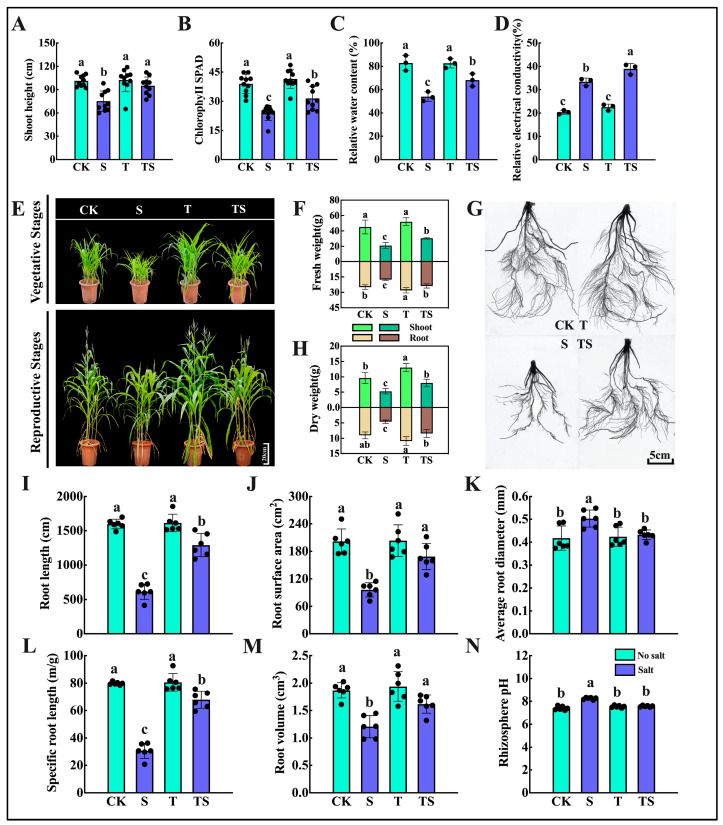
Maize phenotypes in the soil experiment under salt stress and trehalose treatment. Phenotypic photos were recorded during the seedling stage and flowering stage. Measurements included plant height (**A**), chlorophyll content in the aerial parts (**B**), relative water content (**C**), leaf relative conductivity (**D**), seedling and flowering stage phenotypes (**E**), fresh weight (**F**), root system phenotype (**G**), dry weight (**H**), root length (**I**), root surface area (**J**), average root diameter (**K**), specific root weight (**L**), root volume (**M**), and rhizosphere soil pH (**N**). Note: date showing the means ± standard deviation (SD). ns: no significant difference. Lowercase letters: using the one-way ANOVA (Turkey, Duncan), indicating significant differences (*p* < 0.05) between CK (the control), S (the salt stress), 10T (the application of trehalose under normal nutritional) and 10TS (the application of trehalose under salt stress) (*n* = 6).

During the soil culture experiments, the phenotypic records of the maize (Figure 5E,G) and the sampling of seedling leaves for physiological indicator measurements revealed trends similar to those observed in the hydroponic experiments. Firstly, the fresh and dry weights of the maize plants per pot (Figure 5F,H) were significantly inhibited by salt stress, reaching only 0.5 times those of the control, while the application of trehalose broke this suppression, increasing to 0.8 times those of CK. Relative electrical conductivity (Figure 5B), leaf relative water content (Figure 5C), and root growth parameters (Figure 5I–M) exhibited similar trends. Salt stress also caused damage to the cell membranes of the soil-cultured maize leaves, resulting in a significant increase in relative electrical conductivity (Figure 5D).

**Figure 6:**
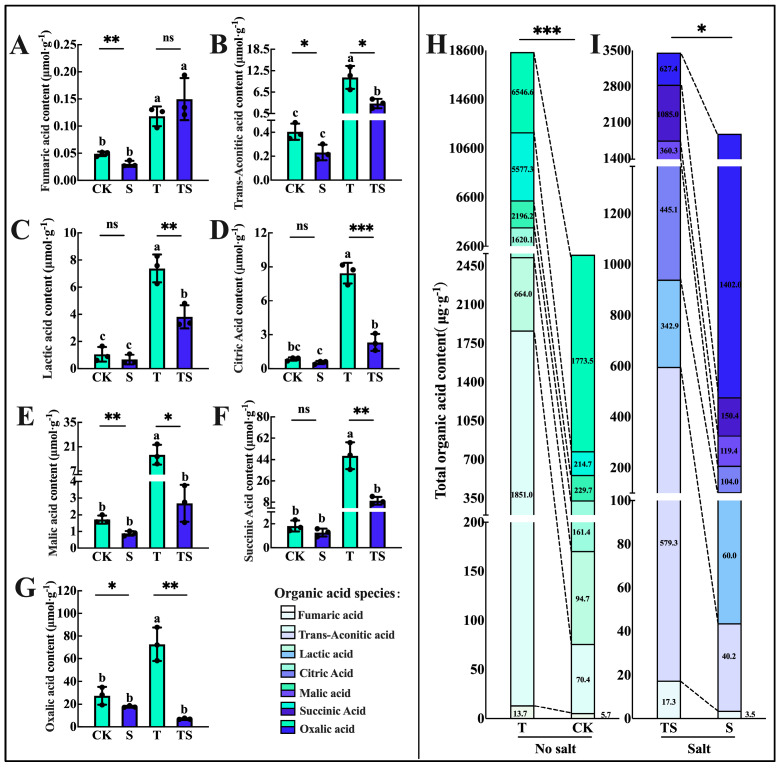
Organic acid content secreted by maize roots in the soil experiment under salt stress and trehalose treatment. The contents of fumaric acid (**A**), aconitic acid (**B**), lactic acid (**C**), citric acid (**D**), maleic acid (**E**), malic acid (**F**), oxalic acid (**G**), total organic acid content secreted by roots under normal conditions (**H**), and total organic acid content secreted by roots under salt stress (**I**) were measured. The data were presented as means ± standard deviation (SD) from three replicates. ns: no significant difference. In panels (**A**–**G**), different letters indicate significant differences (*: highly significant difference at * *p* < 0.05, ** at *p* < 0.01, and *** at *p* < 0.001) between the four treatments using the *t*-test. Different asterisks indicate the significance levels of differences between CK (the control) and S (salt stress), or T (the application of trehalose under normal conditions) and TS (the application of trehalose under salt stress) using the one-way ANOVA (Turkey, Duncan). In panels (**H**,**I**), the contents of various organic acids are marked within the bar chart, and asterisks indicate the significance levels of differences between CK and T, and between S and TS (*p* < 0.05, *n* = 3).

Notably, the pH of the rhizosphere soil under salt stress differed significantly from the other treatments, exhibiting weak alkalinity. Therefore, we measured and analyzed the organic acids secreted by the roots. The addition of trehalose significantly influenced the secretion of seven measured rhizosphere organic acids (fumaric acid, trans-aconitic acid, lactic acid, citric acid, butyric acid, and oxalic acid) (Figure 6A–G). The total amount of organic acids secreted by the maize roots with trehalose application reached 7.44 times that of the control group (Figure 6H). However, salt stress inhibited the secretion of organic acids, reducing it to 0.68 times that of the control group (Figure 6I). Importantly, the application of trehalose under salt stress alleviated this inhibition to some extent, allowing for the secretion of organic acids from the maize roots to reach 1.4 times that of the control group.

### 2.6. Salt Stress and Trehalose-Induced Transcriptome Restructuring in Maize

This study compared the transcriptomic changes in maize leaves under four different treatments (see Section 4). Each treatment included three biological replicates (a total of 12 samples) for analysis. The maize samples from different treatments collectively produced 2.35 billion high-quality reads (approximately 49 million reads per sample). In the RNA-seq analysis, gene expression levels were estimated by counting the sequencing reads mapped to genomic regions or gene exonic regions. The read counts were proportional to the true expression levels of the genes and positively correlated with gene length and sequencing depth.

The DESeq method was used for analysis, and, to identify significantly differentially expressed genes, the filtering criteria were set as *Q* value < 0.05 and |FoldChange| > 2. Using the Spearman correlation coefficient (SCC), hierarchical clustering and principal component analyses (PCA) were conducted based on the average transcripts per million (TPM) values of all expressed genes across the 12 samples. The PCA of the transcriptomic data revealed a clear separation among the maize samples under the different treatments, indicating that salt stress stimulated the re-encoding of the maize transcriptome. Additionally, a distinct separation was observed between the S and 10TS treatments, suggesting that trehalose effectively alleviated salt stress in the maize.

**Figure 7:**
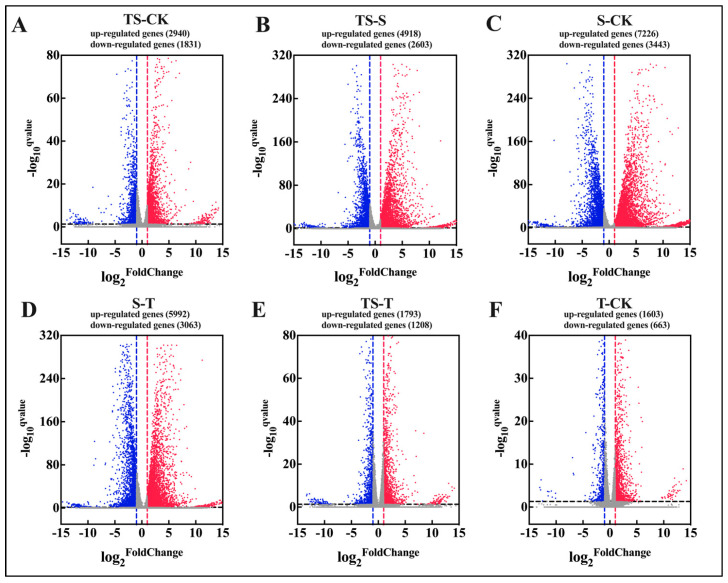
Volcano plot of differentially expressed genes (the *x*-axis represents the fold change in the expression levels of differentially expressed genes, and the *y*-axis represents the logarithm of the *p*-value from the differential expression analysis. The number of upregulated and downregulated genes between treatment groups and experimental groups in pairwise comparisons (**A**–**F**) (CK: the control, S: salt stress, T: the application of trehalose under normal conditions, TS: the ap-plication of trehalose under salt stress.) Red points indicate upregulated genes, blue points indicate downregulated genes, and gray points indicate genes with no significant differential expression) (*Q* value < 0.05, *n* = 3).

To identify differentially expressed genes (DEGs) and further reveal the molecular responses of maize to trehalose under salt stress, a total of 2567 DEGs were identified between the CK and S groups. In the 10TS group, 2553 DEGs were identified. Compared with the S group, the number of activated genes was reduced after the application of exogenous trehalose, indicating that the maize exhibited a certain degree of salt tolerance under this treatment. The treatment with trehalose alone also showed a number of transcriptional differences compared from CK, suggesting that trehalose may promote maize growth and enhance stress resistance by activating the expression of specific genes. Under the conditions of FDR ≤ 0.001, upregulated genes were defined as having log2 FC ≥ 1, while downregulated genes were defined as having log2 FC ≤ 1. This led to an analysis of the expression of unigenes corresponding to genes in the maize leaves under the control and experimental treatments (Figure 7).

### 2.7. Functional Classification of Differentially Expressed Genes (DEGs)

The DEGs that were filtered according to the conditions of |log2 Fold Change| ≥ 1 and FDR < 0.05 for all treatments were subjected to a Gene Ontology (GO) functional analysis (Figure S1). The GO enrichment analysis revealed the gene functions induced by salt stress and trehalose. Under salt stress, the most significantly enriched GO terms in the maize leaves were related to oxidoreductase activity and transmembrane transport protein activity. In the presence of trehalose under salt stress, oxidoreductase activity and thylakoid activity in the maize leaves were the most significantly enriched GO terms compared with the control group, while thylakoid activity and chloroplast activity were the most significant compared with the salt stress group alone.

Additionally, a KEGG enrichment analysis was performed to categorize these DEGs, revealing their potential functions in maize’s response to salt stress and trehalose. Under salt stress, pathways related to photosynthesis, carbon fixation in photosynthesis, sucrose metabolism, and cytochrome P450 metabolism were enriched. In the salt stress samples treated with trehalose, significant enrichment was observed in pathways related to plant hormone signal transduction, the MAPK signaling pathway, photosynthesis, and glycolysis.

Notably, in the CK and S groups without trehalose application, the significantly enriched pathways were mostly related to photosynthesis, MAPK signal transduction, and amino acid metabolism. In contrast, the 10T and 10TS groups with trehalose application showed enrichment in various carbon metabolism pathways. These results provide a transcriptional overview of the functional pathways induced by trehalose treatment to enhance salt tolerance in maize. The following sections present further discussions on the regulatory gene expression in differentially compared transcriptional pathways.

### 2.8. Transcriptional Differences in Sugar Metabolism Under Salt Stress and Trehalose Treatment

All genes related to trehalose and glycolysis in maize were identified to assess the expression changes of trehalose metabolism and glycolysis genes under different treatments (Figure S2). These included trehalose-6-phosphate synthase (T6P), hexokinase (HK), 6-phosphofructokinase (PFK9), pyruvate kinase (PK), and aldolase (ALDO) (see Supplementary Materials). Under salt stress, the genes regulating PGK (5 genes), GPI (3 genes), gapN (1 gene), and gpmB (2 genes) exhibited only minimal expression (log2|TPM| < 1) compared with the control. The 10T and 10TS treatments did not lead to a significant differential expression of these genes. In contrast, under salt stress, most genes associated with trehalose synthesis and glycolysis were upregulated. The genes that were most significantly upregulated included HK (8 genes), phosphofructokinase PFP (6 genes), PK (12 genes), and ALDO (8 genes). The application of trehalose under salt stress slightly further increased the expression of these genes, with fold increases of 1.4, 1.1, 1.6, and 1.3, respectively, compared with the salt stress treatment alone.

It is noteworthy that the 10T treatment suppressed the expression of two trehalose-6-phosphate phosphatase (TPP) genes. Additionally, under the CK and 10T conditions, there was one non-expressed trehalose-6-phosphate synthase (TPS) gene, which was significantly downregulated under both the S and 10TS conditions. However, the expression of other genes related to trehalose synthesis and metabolism was not significantly affected. Under salt stress, the expression levels of most genes associated with trehalose synthesis and metabolism (TPP, TPS, TRE) were elevated. Especially under salt stress, the application of trehalose further enhanced the expression of these genes.

### 2.9. Transcriptional Differences in Abscisic Acid Signaling and MAPK Pathway Under Salt Stress and Trehalose Treatment

Under salt stress, the signaling molecules from the trehalose synthesis pathway were identified as candidate pathways for gene annotation through the regulation of plant hormone signaling and the MAPK signaling cascade (Figure S3). In the presence of salt stress (S), the expression of MPK1_2 in the maize leaves was significantly upregulated. In contrast, the genes encoding PP2C were significantly downregulated in response to salt stress. Moreover, salt stress significantly upregulated the genes for abscisic acid receptors (PYR/PYL) and SnRK2s (SNF1-related protein kinases), while downregulating the expression of type 2C protein phosphatases (PP2C). Three transcription factors and ABRE-binding factor (ABF) genes were also upregulated under the salt stress treatment. These results suggest that maize responds to salt stress through the overexpression of PYR/PYL, which subsequently inhibits PP2C and releases SnRK2s, thereby activating downstream ABF.

Additionally, salt stress significantly upregulated the MAPKKK17/18 kinases activated by abscisic acid (ABA). Notably, the gene for mitogen-activated protein kinase (MPK6) showed a slight upregulation, indicating that salt stress induced the accumulation of H_2_O_2_ in the maize, which further impacted the expression of catalase 1 (CAT1). After the trehalose treatment, CAT1 expression was significantly upregulated under salt stress, while MPK1_2, PYR/PYL, and SnRK2s were slightly upregulated compared with the salt stress treatment alone. Additionally, the trehalose treatment resulted in a decrease in MPK6 expression.

### 2.10. Transcriptional Changes in Photosynthesis Under Salt Stress and Trehalose Treatment

**Figure 8:**
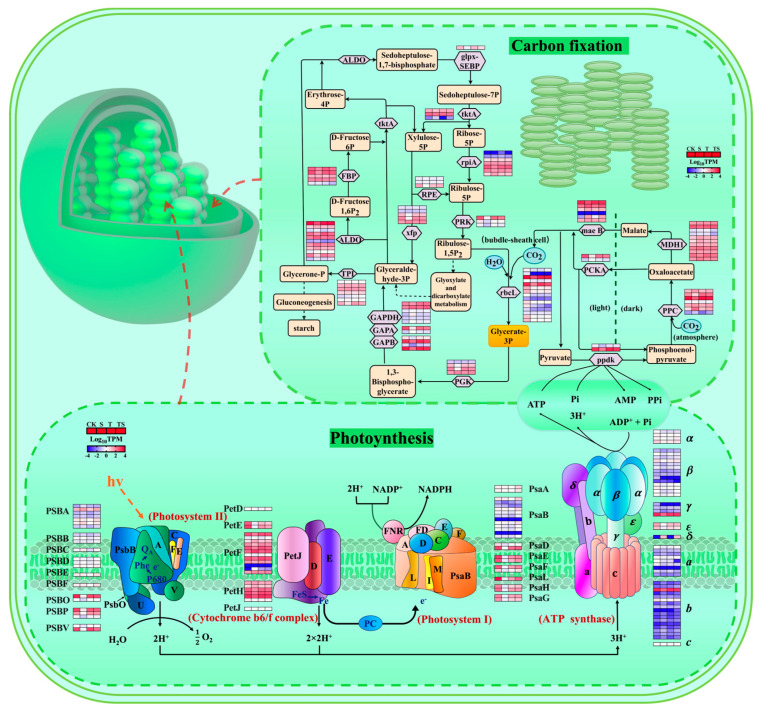
Transcriptional differences in photosynthesis in maize leaves under salt stress and trehalose treatment. The colored blocks represent log2 TPM values (CK: the control, S: salt stress, T: the application of trehalose under normal conditions, TS: the application of trehalose under salt stress). Red and blue indicate significantly upregulated and downregulated genes, respectively (log2 |TPM| ≥ 1, *Q* value < 0.05, *n* = 3). For enzyme reactions, the direction of arrows indicates the order of signal transduction. In the dark reaction, transcription proteins and transcription factors are represented by orange boxes, metabolites by orange squares, and key metabolites by a darker orange. PPC: phosphoenolpyruvate carboxylase; ppdk: pyruvate, orthophosphate dikinase; PCKA: phosphoenolpyruvate carboxykinase; MDH1: malate dehydrogenase; mae B: malate dehydrogenase; pgk: phosphoglycerate kinase; GAPA: glyceraldehyde 3-phosphate dehydrogenase; GAPB: glyceraldehyde-3-phosphate dehydrogenase (NAD(P)^+^); GAPDH: glyceraldehyde-3-phosphate dehydrogenase; TPI: triosephosphate isomerase; PRK: phosphoribulokinase; rbcL: ribulose-bisphosphate carboxylase large chain; xfp: xylulose-5-phosphate/fructose-6-phosphate phosphoketolase; rpi A: ribose 5-phosphate isomerase A; tktA: transketolase; glpx-SEBP: fructose-1,6-bisphosphatase II/sedoheptulose-1,7-bisphosphatase; ALDO: fructose-bisphosphate aldolase, class I; FBP: fructose-1,6-bisphosphatase I; RPE: ribulose-phosphate 3-epimerase. Photosystem II (PSII) proteins (PsbA, PsbB, PsbC, PsbD, PsbE, PsbF, PsbO, PsbP, and PsbV); PSI proteins (PsaA, PsaB, PsaC, and PsaD); cytochrome b6 complex (PetD, PetE, PetF, PetH, and PetD); F-type ATPase proteins (ATPaseα, β, γ, ε, δ; a, b and c).

The data for both the light and dark reactions were compiled and analyzed (Figure 8). In this study, during the dark reaction of photosynthesis under salt stress, the expression of genes related to phosphoenolpyruvate carboxylase (PPC) was downregulated, likely due to stomatal closure and reduced CO_2_ uptake under salt stress. However, the expression of NADP-malate dehydrogenase (MDH1) remained unaffected. Salt stress downregulated the expression of pyruvate phosphate dikinase (PPDK), inhibiting the conversion of pyruvate to phosphoenolpyruvate. Additionally, the expression of key enzyme synthesis genes such as glyceraldehyde-3-phosphate dehydrogenase (GAPA), phosphoglycerate kinase (GAPB), fructose-6-phosphate phosphoketolase (xfp), and ribulose-5-phosphate kinase (PRK) was also downregulated.

However, the application of trehalose under salt stress alleviated these inhibitory effects by upregulating the expression of these enzyme synthesis genes, thus promoting the dark reaction in the maize leaves (Figure 8). Under salt stress, the expression of genes encoding photosystem I (PSI) (PsaA, PsaB, PsaE, PsaF, PsaL, PsaH, and PsaG) and II (PSII) proteins (PsbA, PsbB, PsbC, PsbD, PsbE, PsbF, PsbO, PsbP, and PsbV) was downregulated, along with the cytochrome b6f complex proteins (PetD, PetE, PetF, PetH, and PetJ) and F-type ATPase proteins (ATPase-α, β, γ, ε, δ; a and b). These results indicate that salt stress severely inhibited the light reaction in the maize.

The effect of trehalose on the photosynthetic process under salt stress showed different outcomes. Trehalose significantly upregulated the expression of genes encoding PSI, PSII, and F-type ATPase proteins in the maize. Notably, the application of trehalose also enhanced CO_2_ fixation in the maize leaves, promoting water use efficiency and energy production, maximizing the advantage of the C4 plants’ high efficiency in utilizing light energy. These findings suggest that trehalose improves maize’s salt tolerance by effectively regulating genes involved in the photosynthetic pathway.

### 2.11. Temporal and Spatial Changes in Trehalose and Abscisic Acid Metabolism-Related Gene Expression

**Figure 9:**
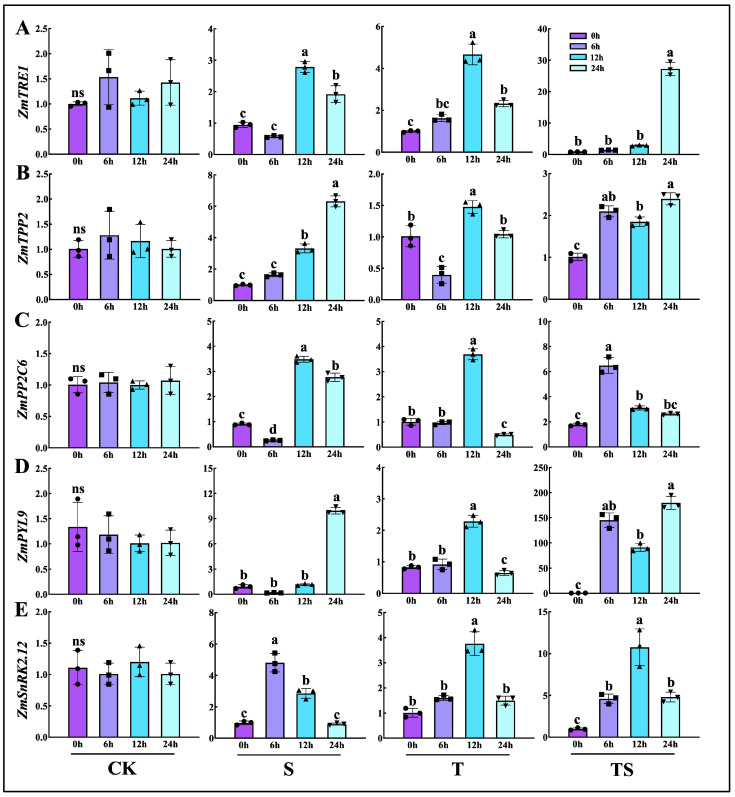
Relative expression levels of genes at different time points under hydroponic conditions. Sampling time points were 0 h, 6 h, 12 h, and 24 h after the onset of salt stress. The relative expression levels of *ZmTRE1* (**A**), *ZmTPP2* (**B**), *ZmPP2C6* (**C**), *ZmPYL9* (**D**), and *ZmSnRK2.12* (**E**) were measured. Note: date showing the means ± standard deviation (SD). ns: no significant difference. Different letters indicate significant differences between the four treatments (CK: the control, S: salt stress, T: the application of trehalose under normal conditions, TS: the application of trehalose under salt stress) (*p* < 0.05, *n* = 3; Duncan test).

To further verify the transcription factor levels under salt stress in maize and explore the relationship between trehalose and abscisic acid (ABA), five genes related to trehalose and ABA receptors were selected from the transcriptome sequencing results for a relative expression analysis. Maize samples from the hydroponic experiment were collected at different time points, and qRT-PCR was performed after constructing cDNA libraries. After 24 h of salt stress treatment (Figure 9), the expression levels of *ZmTRE1*, *ZmTPP2*, *ZmPYL9*, and *ZmPP2C6* were all upregulated, while *ZmSnRK2.12* showed a significant upregulation between 6 and 12 h. A similar trend was observed in the maize subjected to salt stress combined with trehalose treatment, with even more pronounced changes. However, it is noteworthy that the trehalose synthesis-related gene *ZmTPP2* exhibited a different expression pattern under this combined treatment compared with salt stress alone. Genes related to trehalose metabolism showed changes in the trehalose-only treatment, but the expression of ABA-related genes did not show significant differences from the CK.

**Figure 10:**
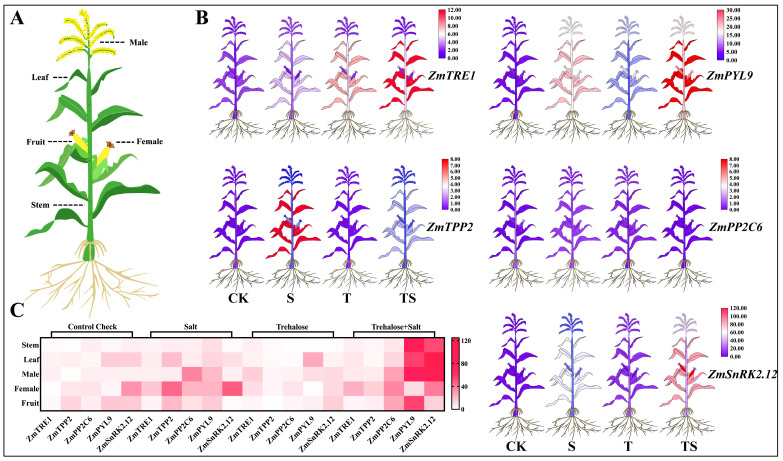
In the soil cultivation experiment, the expression analysis of maize genes *ZmTRE1*, *ZmTPP2*, *ZmPP2C6*, *ZmPYL9*, and *ZmSnRK2.12* in different tissues under various treatments was conducted (*p* < 0.05, *n* = 3) (CK: the control, S: salt stress, T: the application of trehalose under normal conditions, TS: the application of trehalose under salt stress). A plant simulation cartoon (**A**) was used to create two forms of gene expression heat maps: a heat map (**B**) and a matrix heat map (**C**), showing the expression levels of the above genes in stem, leaf, female, male, and fruit. In the cartoon heat map, red indicates high expression levels, while blue indicates low expression levels. In the matrix heat map, red indicates high expression levels, and white indicates low expression levels.

Gene expression patterns in various plant tissues can reveal their biological functions during growth and development. In this study, qRT-PCR data from soil-grown maize were used to analyze the expression patterns of the selected genes in maize tassels, ears, fruits, stems, and leaves under different treatments (Figure 10). The results showed that the five genes exhibited various levels of expression in different tissues and under different treatments.

The signal-regulating genes of the abscisic acid (ABA) receptor, including *ZmPYL9*, *ZmPP2C6*, and *ZmSnRK2.12*, displayed significant differences in expression under salt stress (S, TS), while there was no noticeable difference under normal conditions (CK, T) (Figure 10C). However, trehalose synthesis and metabolism-related genes (*ZmTRE1* and *ZmTPP2*) showed distinct expression patterns under the trehalose treatment and salt stress, with the expression levels following the order of TS > S > T > CK (Figure 10B). This might be due to trehalose acting as an osmoprotectant, accumulating under stress conditions to protect plants from damage. *ZmPYL9*, *ZmTRE1*, and *ZmSnRK2.12* were expressed in various maize tissues, while *ZmPP2C6* exhibited relatively low expression across different tissues. *ZmSnRK2.12* had the highest expression in the female flowers, followed by *ZmTRE1* and *ZmPYL9*. Similarly, *ZmPYL9* was the most highly expressed in the female flowers, followed by *ZmTRE1* and *ZmTPP2*. These genes might play an active role in flower organ development through the trehalose regulatory pathway. In the maize stems and leaves, *ZmSnRK2.12*, *ZmTRE1*, *ZmTPP2*, and *ZmPYL9* were expressed at higher levels, whereas *ZmPP2C6* had lower expression. This suggests that these genes are more likely to regulate photosynthesis and cell division in these tissues under salt stress. Moreover, *ZmPYL9*, *ZmTRE1*, *ZmSnRK2.12*, and *ZmTRE1* were significantly expressed in the maize fruit, indicating their potential roles in regulating stress tolerance during fruit development and influencing fruit growth.

## 3. Discussion

Maize is an important food and economic crop worldwide. However, salt stress severely harms maize growth and yield. Salt stress disrupts the internal osmotic balance of plants, affecting their ability to absorb and transport nutrients and ions normally, which can lead to ion toxicity [37] and even the collapse of the antioxidant system, causing changes in gene expression and hormone levels [38]. Many studies indicate that salt stress inhibits root growth, making it difficult for plants to absorb nutrients [39]. However, the plant’s response to salt stress is a process of adaptation through physiological metabolism and gene expression. Metabolites, as the ultimate products of gene expression and biochemical reactions, provide a direct link between genotype and phenotype. By combining physiological, biochemical, and transcriptomic analyses, we can comprehensively study the gene metabolism networks related to plant responses to abiotic stress [40]. For a long time, most improvements in maize salt tolerance have been achieved through molecular breeding, but this method is time-consuming and inefficient. The trehalose signaling pathway is not linear; rather, it is a complex network that dynamically interacts with other signaling pathways, such as salt signaling and plant hormone signaling. The cross-talk of trehalose signaling with other sugar and hormone signaling pathways plays a role in regulating basic physiological functions and salt tolerance [41]. This experiment uses foliar application of trehalose to explore the pathways through which trehalose enhances salt tolerance in maize. Analyzing the molecular mechanisms by which trehalose regulates salt tolerance can help plant scientists and breeders improve crop yield by manipulating key regulatory components [42].

### 3.1. Salt Stress-Induced Carbon Loss Accelerates Maize Senescence

The homeostasis of endogenous sugars in plants aids in energy metabolism and membrane stability, and the transport and distribution of sugars directly affect plant growth and development [41]. The interruption of the tricarboxylic acid cycle caused by salt stress may be due to the blockage of intermediate synthesis metabolism, reducing the supply of energy and nutrients during early germination, thereby inhibiting seed germination [43]. Trehalose alleviates temperature stress by affecting the central carbon metabolism in mushrooms [44]. Trehalose protects the biomembrane system of wheat under temperature stress, promoting carbohydrate metabolism and signaling, enhancing TCA cycle activity, and thus regulating heat tolerance in wheat [45]. ABA may significantly increase rice seed setting rates under temperature stress through trehalose metabolism and ATP consumption [46]. T6P inhibits sucrose non-fermenting related protein kinase 1 (SnRK1), which is a global integrator of energy balance. When stress responses lead to decreased energy levels, SnRK1 is activated and triggers the induction or repression of about 1000 genes, shifting from anabolic to catabolic metabolism, promoting plant survival rather than growth [47]. Trehalose-6-phosphate synthase 1 (TPS1) is known to promote trehalose synthesis and further influence plant thermotolerance through its regulatory role in carbon distribution and sucrose balance. Trehalose treatment reduces the activity of sucrose hydrolases, such as sucrose synthase (invertase) and sucrose phosphate synthase, which can improve energy levels and membrane stability under low-temperature conditions by regulating soluble sugar metabolism, maintaining fruit quality [48]. In yeast cells, T6P inhibits hexokinase activity and alters the rate of glycolysis [49]. In this study, salt stress reduced the levels of soluble sugars under salt stress (Figure 2). Transcriptomic data indicate that salt stress promotes the process of glycolysis, accelerating the depletion of energy substances. Under trehalose treatment, the transcription levels of key transcription factors involved in sugar metabolism are elevated, generating energy to enhance metabolic levels. The sucrose deficit required for growth is replenished through the metabolic products of the trehalose synthesis pathway.

### 3.2. Trehalose Can Alleviate the Inhibition of Photosynthesis

Photosynthesis is an important physiological process that regulates plant development and responses to abiotic stress [50]. Salt stress increases oxidative damage in plants, leading to an elevated production of reactive oxygen species (ROS). These ROS can damage photosynthetic pigments, further disrupting photosynthetic mechanisms and stomatal regulation. After the application of exogenous bioregulators, the photosynthetic leaf area of maize increased, the rate of leaf senescence slowed, and dry matter accumulation after flowering increased, resulting in higher grain weight and spikelet number, ultimately enhancing maize yield [51]. In our study, the supplementation of trehalose enabled maize to achieve higher photosynthetic efficiency under salt stress. Trehalose can regulate the expression levels of multiple differentially expressed genes related to photosynthetic pathways. It can induce several photosynthetic genes belonging to the photosystems (PsaA, PsaB, PsaE, PsaF, PsaL, PsaH, PsaG, PsbA, PsbB, PsbC, PsbD, PsbE, PsbF, PsbO, PsbP, and PsbV) and the electron transport chain (PetD, PetE, PetF, PetH, and PetJ). Additionally, trehalose can also regulate the Calvin cycle process in photosynthesis, affecting the transcription levels of genes related to carboxylation, reduction, and ribulose bisphosphate regeneration. The GO enrichment results indicate that trehalose protects the ultrastructure of thylakoid chloroplasts and certain peptides under salt stress, thereby improving the absorption, utilization, and distribution efficiency of light energy in maize plants. The relative chlorophyll content in the trehalose-sprayed plants was higher, indicating that trehalose is beneficial for the gene expression of maize’s photosynthetic mechanisms and supports the signaling pathways related to chlorophyll biosynthesis. This aligns with the findings of a previous study which showed that trehalose increased the maximum electron transport chain efficiency (rETRmax) and light energy utilization efficiency, positively impacting tomato growth under salt stress [52].

### 3.3. Trehalose Promotes Organic Acid Secretion in Roots, Regulating the Root Ecological Environment and Ion Homeostasis to Alleviate Salt Stress

The root system plays an important role throughout the entire growth period of maize. Plant roots exist in the soil, providing the water and minerals necessary for growth. However, high-pH conditions can cause severe damage and death to root cells, limiting mineral absorption [53]. Healthy roots can maintain mineral uptake and protect the plant [15]. K^+^ is essential and the most abundant cation in plants, while Na^+^ often coexists with K^+^, being beneficial yet a major contributor to salt-alkali toxicity. K^+^ absorbed by the roots is transferred to the aerial parts through the xylem, with 40–90% of K^+^ returning to the roots through the phloem. K^+^, along with sugars and other nutrients, regulates the transport of photosynthetic products from “source” to “sink”, affecting grain yield [54]. Na^+^ toxicity is one of the important factors inhibiting plant growth. Increased Na^+^ assimilation disrupts the balance of K^+^, Ca^2+^, and Mg^2+^, leading to nutrient deficiency and ion imbalance in plant tissues [55]. Improving the ecological environment in the rhizosphere under salt stress may enhance plant salt tolerance.

Sugars are important structural components and energy substrates for plants, and they also act as signaling molecules regulating root functions. Their transport within the plant is crucial for root morphology. Exogenous sugar can influence root hair growth and root gravitropism [56]. In this study, trehalose was found to promote the secretion of organic acids from maize roots, especially oxalic acid, thus improving the physicochemical properties of the rhizosphere soil and stabilizing the transport homeostasis of sodium and potassium ions, alleviating ion toxicity due to salt stress on maize. The application of trehalose enhanced the growth of maize roots, including root surface area, root volume, and root biomass, leading to improved nitrogen and phosphorus uptake and accumulation, ensuring robust growth and increased salt tolerance. The increase in the effective surface area of the roots plays a key role in facilitating water and nutrient absorption from the soil [57]. This is consistent with previous studies indicating that trehalose treatment promotes root development and organic matter content in tomato seedlings while maintaining a lower Na^+^/K^+^ ratio, further enhancing the plant’s adaptation to salt stress [17]. However, the mechanisms by which trehalose improves the rhizosphere soil microenvironment through organic acids to further affect sodium and potassium ion transport still require further investigation and analysis.

### 3.4. Abscisic Acid Signaling and MAPK Signaling Regulate the Antioxidant System to Mitigate Oxidative Damage in Maize

It is well known that oxidative stress signaling and reactive oxygen species detoxification are important components of salt stress tolerance mechanisms [58]. ABA is a central regulatory factor in stress responses, and its levels and signaling components are closely related to plant salt tolerance. The dehydration-responsive element-binding (DREB) subfamily transcription factors and the MAPK signaling pathway play crucial roles in plant responses to abiotic stress. SOD, POD, CAT, and APX are important antioxidant enzymes in plants; SOD converts superoxide anions to H_2_O_2_, while the other three enzymes are responsible for removing excess H_2_O_2_. The key transcription factor ABF2, downstream of ABA signaling, is activated by TPP and TRE to clear ROS [24]. Similar results indicate that the overexpression of DREB family genes can promote the expression of antioxidant enzyme genes [59]. Trehalose and its derivatives are not only metabolic resources and structural components of plant cells but also possess hormone-like regulatory properties [41].

In this study, salt stress stimulated the production of abscisic acid (ABA) (Figure S3), and, as a result, the mRNA levels of PYL-binding factors, which act downstream of ABA receptors, were downregulated, inhibiting the transcription of PP2Cs genes. This led to an increase in the release of SnRK2s. However, after the exogenous application of trehalose, the inhibition of PP2Cs was alleviated, further increasing the transcription of PYL and SnRK2, thereby enhancing ABA signal transduction. Interestingly, the transcription of the TPP2 gene was suppressed after the exogenous application of trehalose, although TPP is a gene that positively regulates the T6P-ABA signaling pathway [60]. This suggests that in this experiment, exogenous trehalose may not have regulated ABA through its upstream synthesis signals. The improvement in salt tolerance observed in maize may be due to the accumulation of exogenous trehalose in the maize leaves, likely via diffusion, where it is hydrolyzed into glucose to support starch and sucrose metabolism, providing energy substrates. This could counteract the inhibitory effects of salt stress on maize growth. However, this hypothesis requires further experimental validation. The findings of this study demonstrate that trehalose can increase the expression of trehalose metabolism-related genes in maize leaves under salt stress and regulate endogenous ABA synthesis. Additionally, the signal molecules of ABA receptors and the ABA-mediated MAPK signaling pathway can activate leaf antioxidant enzyme activities, which help scavenge excess reactive oxygen species (ROS) and maintain redox homeostasis. This effectively mitigates the growth inhibition and cell membrane damage caused by salt stress in maize plants.

## 4. Materials and Methods

### 4.1. Plant Materials

Maize seeds (*Zea mays* L. cv. Zhengdan985, a local commonly planted cultivar) were disinfected on the surface with 10% hydrogen peroxide (H_2_O_2_) for 10 min, soaked in distilled water at room temperature for 8 h, and then transferred to germination boxes to germinate in the dark at room temperature for 48 h.

### 4.2. Hydroponic Experiment

**Figure 11:**
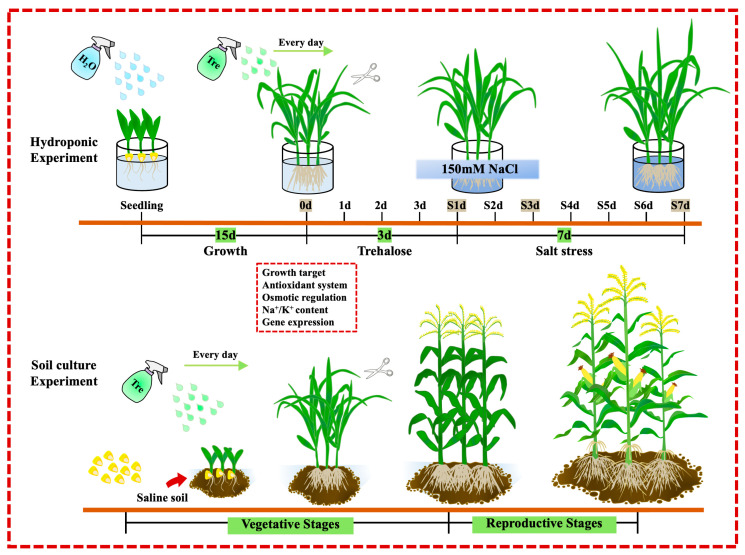
Experimental design diagram. The time points with gray shading represent the sampling times in the hydroponic experiment. Tre: 10 mM trehalose was sprayed twice daily, at 9 a.m. and 9 p.m. A 150 mM NaCl solution was used to simulate salt stress in the hydroponic experiment. Soil containing 0.175% NaCl was used to simulate growth in saline soil.

A hydroponic system was used to simulate salt stress conditions, maintained in a greenhouse with temperatures of 28/22 °C and a light/dark cycle of 16/8 h, at a relative humidity of 65%. Germinating seeds with similar conditions were transferred to plastic buckets containing Hoagland nutrient solution (height × bottom diameter × top diameter = 15 cm × 12 cm × 14 cm) and grown under natural greenhouse light for 25 days, with the solution refreshed every 4 days. On the 15th day, the maize was divided into four treatment groups. The control group grew in Hoagland solution for 25 days. The 10T and 10TS groups received foliar sprays of 10 mM trehalose (3.78 g dissolved in 1 L distilled water. 10 mM is the optimal concentration selected from preliminary experiments.) twice daily until harvest, starting after 15 days. On the 18th day, 150 mM NaCl was added to the hydroponic solution for the salt stress treatments (S and 10TS). Each treatment had 5 replicates, with 15 plants each. The sampling points (Figure 11) were 0d, S1d, S3d, and S7d for phenotypic, physiological, RNA sequencing, and RT-qPCR analyses.

### 4.3. Pot Experiment

In the pot experiment, sieved farmland soil was placed in plastic pots (height × bottom diameter × top diameter = 15 cm × 12 cm × 14 cm) and grown in an outdoor natural environment, maintaining a field water capacity of 80%. The control group received normal irrigation throughout the experiment. The T and TS groups were sprayed twice daily with 10 mM trehalose, ensuring that each leaf surface was covered with droplets. The salt stress experiment was designed as shown in Figure 11. In the soil salt stress treatments, the soil contained 0.175% NaCl. Each treatment had 5 biological replicates with a total of 15 plants; the plants were sampled at the seedling stage for phenotypic and physiological analysis, and different tissues were sampled at various growth stages for an RT-qPCR analysis.

### 4.4. Growth Parameter Measurement

At the designated sampling time points, seedling growth in the experiment was imaged using a high-resolution camera (Nikon, Tokyo, Japan). On the day of harvest, the height of each maize plant was measured from the junction of the root and stem to the top of the plant (cm). Chlorophyll SPAD values were measured using a handheld chlorophyll meter (SPAD-Plus) on four fully expanded leaves at the same position on each plant. The maize was harvested at the specified time points for fresh weight (FW) measurement. The samples were first blanched at 105 °C for 15 min and then dried at 70 °C until a constant weight was reached to determine dry weight (DW). The relative water content (RWC, g DW^−1^) was calculated as (FW − DW)/DW, where FW and DW represent the fresh and dry weights, respectively. After washing the roots, a double-sided scanner (EPSON GT-X900, EPSON, Nagano, Japan) was used to scan them, and images were analyzed using a WinRHIZO image analysis system (WinRHIZO Pro 2009b, Regent Instructions, Quebec, QC, Canada) to determine root morphological parameters, such as total root length (cm), root surface area (cm^2^), average root diameter (mm), and root volume (cm^3^). The specific root length was calculated as the total root length divided by the root dry weight.

### 4.5. Hydrogen Peroxide Measurement

Histochemical staining for H_2_O_2_ in the leaves was conducted using DAB staining [61]. Fresh leaves from the same leaf position were washed with deionized water and completely immersed in a 1 mg/L diaminobenzidine (DAB) solution. The leaves were incubated under light at 25 °C for 2 h. After incubation, the DAB solution was removed, and the leaves were thoroughly rinsed with deionized water 4–5 times to ensure that all residual DAB was removed. The leaves were then immersed in 95% ethanol and boiled in a water bath until fully decolorized. After decolorization, the leaves were observed for staining patterns, and photographs were taken for documentation.

The H_2_O_2_ content was measured using the potassium iodide reduction method [62]. For the determination of the H_2_O_2_ content, 0.2 g of fresh leaves was weighed and homogenized in 0.1% (*w*/*v*) trichloroacetic acid. The homogenate was transferred to a pre-chilled centrifuge tube and centrifuged at 12,000× *g* for 20 min at 4 °C. A 0.5 mL aliquot of the supernatant was mixed with 0.5 mL of 10 mmol·L^−1^ K_3_PO_4_ buffer (pH 7.0) and 1 mL of 1 mol·L^−1^ KI. The mixture was incubated in the dark for 15 min, and the absorbance of the supernatant was measured at 390 nm.

### 4.6. Measurement of Antioxidant Enzyme Activity and Malondialdehyde (MDA)

A 0.5 g sample of fresh maize leaves from each treatment was taken and mixed with 2.5 mL of phosphate-buffered saline (PBS, pH 7.0). The mixture was ground in an ice bath and then centrifuged at 15,000 rpm for 15 min. A portion of the supernatant was collected, appropriately diluted, and used for enzyme activity assays. Superoxide dismutase activity (SOD) was determined by measuring the inhibition of nitroblue tetrazolium (NBT) reduction in a riboflavin environment. The following components were added to a 5 mL centrifuge tube: 4.05 mL of PBS, 0.3 mL of Met solution, 0.3 mL of NBT solution, and 0.3 mL of riboflavin solution. Finally, 0.3 mL of enzyme solution was added. These tubes were placed under light conditions for a 20 min reaction. Subsequently, absorbance was measured at 560 nm using a spectrophotometer (INESA 752N, Shanghai, China). Peroxidase activity (POD) was based on the oxidation of guaiacol. A 5 mL centrifuge tube was filled with 1 mL of 0.3% H_2_O_2_, 0.95 mL of 0.2% guaiacol, and 1 mL of pH 7.0 PBS. Finally, 0.05 mL of enzyme solution was added to initiate the reaction. The rate of increase in OD at 470 nm was recorded. An increase of 0.01 in OD per minute was defined as one activity unit (U/g FW). A 5 mL centrifuge tube was filled with 1 mL of 0.3% H_2_O_2_ and 1.95 mL of H_2_O. Finally, 0.05 mL of enzyme solution was added to initiate the reaction. The rate of decrease in OD at 240 nm was measured. A decrease of 0.01 in OD per minute was defined as one catalase (CAT) activity unit (U/mg). The measurement of ascorbate peroxidase (APX) activity was determined by observing the oxidation of ascorbate in a hydrogen peroxide environment [63]. A mixture of 300 μL of crude enzyme solution and 3 mL of reaction solution (50 mM phosphate buffer, pH 7.0, and 0.5 mM AsA) was prepared. Then, 20 μL of 0.05% H_2_O_2_ was added and quickly mixed thoroughly. The absorbance at 290 nm was measured every minute, and the decrease in absorbance was used to determine ascorbate peroxidase (APX) activity.

To measure the MDA content, fresh leaves were extracted with 1% trichloroacetic acid, and the supernatant was mixed with 0.5% thiobarbituric acid (20% trichloroacetic acid). The reaction mixture was then incubated at 96 °C for 25 min, and the absorbance of the cooled samples was recorded at 532 nm [64].

### 4.7. Measurement of Osmotic Substances

Leaves were incubated in distilled water at 25 °C, and the conductivity (C1) was recorded. After that, the samples were steam-pressed at 120 °C for 20 min to obtain C2. The relative electrical conductivity (REC) was calculated as REC (%) = [1 − (C1/C2)] × 100% [63].

Soluble sugar and soluble protein were determined by using Chen’s method [17]. A 0.1 g sample of maize leaf tissue was placed in a 25 mL graduated test tube, and 25 mL of deionized water was added. The tube was incubated in a boiling water bath at 100 °C for 1 h. The mixture was filtered in a 100 mL volumetric flask, and the volume was adjusted to 100 mL with deionized water. A 0.5 mL aliquot of the extracted solution was mixed with 0.5 mL of deionized water and 5 mL of anthrone reagent. After the reaction was completed, the absorbance was measured at 620 nm using a spectrophotometer. A 0.1 mL aliquot of the maize leaf extract was taken and mixed with 5 mL of Coomassie Brilliant Blue G-250 reagent. After the reaction was completed, the absorbance was measured at 595 nm using a spectrophotometer.

For the detection of proline (Pro) in the maize leaves, the method proposed by Mukarram, et al. [65] was used. A 0.5 g sample of maize leaf tissue was weighed, chopped into small pieces, and placed in a stoppered test tube. Then, 2 mL of deionized water, 2 mL of glacial acetic acid, and 4 mL of acidic Ninhydrinhydrate were added. The mixture was shaken thoroughly and heated in a boiling water bath for 60 min to allow for color development. After heating, the tube was cooled to room temperature, and 4 mL of toluene was added. The mixture was vigorously shaken to extract the red product. The absorbance of the toluene extract layer was measured at 520 nm using a spectrophotometer (INESA 752N, Shanghai, China).

### 4.8. Measurement of Na^+^ and K^+^

According to the method by Tang, et al. [66], Dried maize tissue was ground into a fine powder, and 0.3 g of the sample was weighed into a digestion tube. The sample was digested using an externally heated H_2_SO_4_-H_2_O_2_ digestion method. The resulting digest was analyzed using a flame photometer to determine the concentrations of Na^+^ and K^+^ (mg/g) (Corning, Cambridge, UK).

### 4.9. Measurement of Rhizosphere Physiological Parameters

At harvest, the roots were gently shaken to remove loosely attached soil and immersed in a beaker containing 50 mL of 0.2 mM CaCl_2_ solution. After shaking for 1 min to remove more tightly adhered soil, the filtered extract was defined as rhizosphere extract. The pH of the rhizosphere was measured using a pH meter. The types and contents of organic acids in the rhizosphere exudate were determined following Shen’s method [67] using high-performance liquid chromatography (HPLC). The analysis was conducted with a Sonoma C18(2) column (25 cm × 4.6 mm, 5 μm). A sample injection volume of 20 μL was used. The mobile phases consisted of the following: A, phosphate buffer; B, acetonitrile; C, high-purity water; and D, methanol. The column temperature was maintained at 31 °C, with a flow rate of 1.0 mL/min.

### 4.10. RNA-seq Library Construction and Transcriptome Analysis

All plants exhibit corresponding stress responses under physiological dehydration conditions. Fully expanded leaves from four treated maize plants (3 replicates) were selected for RNA-Seq analysis. Twelve libraries (4 treatments-3 replicates) were sequenced using Novaseq 6000 (Illumina) software (LC Sciences, Houston, TX, USA), generating paired-end sequences of 150 nucleotides. Following the manufacturer’s instructions, total RNA was extracted from the samples using the Total RNA Extractor kit (Sangon, Shanghai, China). RNA concentration was assessed using an SMA4000 micro-spectrophotometer (Merinton, Beijing, China). RNA quality was evaluated with an Agilent 2100 Bioanalyzer (Agilent Technologies, Santa Clara CA, USA), and RNA integrity and genomic contamination were examined using agarose gel electrophoresis [68]. Subsequently, the libraries were sequenced on an Illumina Novaseq 6000 (Illumina, San Diego, CA, USA) system, generating 150 bp paired-end reads [69].

### 4.11. RT-qPCR Analysis Verification

Genes with significant differences in transcription levels identified in this study were selected for further analysis. Total RNA was extracted from the maize leaves, stamens, pistils, and stems using an RNAiso Kit (D326S, Thermo Fisher, Waltham, MA, USA). RNA samples were treated with DNase to remove genomic DNA, and RNA concentration and quality were assessed using an SMA4000 micro-spectrophotometer (Merinton, Beijing, China) and 1% agarose gel electrophoresis. The total RNA was used as a template for cDNA synthesis with a UEIris II RT-PCR system and a First-Strand cDNA Synthesis (with dsDNase) kit (R2028, US Everbright^®^ Inc., Hayward, CA, USA). Gene-specific primers were designed using Primer Express (v3.0), and the PCR program and system are referenced in Table S1. The maize actin gene was used as an internal control, and qRT-PCR analysis was performed using the 2×Fast Super EvaGreen qPCR Master Mix (S2008, US Everbright^®^ Inc. Hayward, CA, USA), with the qPCR program system referenced in Table S3. Each biological treatment was repeated three times, and data were calculated using the 2−ΔΔCt method to determine the relative changes in gene expression levels [69]. RT-qPCR heatmaps were generated using TBtools toolkit (version 2.1) [70].

### 4.12. Statistical Analysis

In this study, each indicator and gene expression are represented as the mean ± standard deviation (SD) from three independent experiments. The samples treated with T, S, and TS were compared with the control samples, except for the untreated group. A *p*-value of ≤0.05 or ≤0.01 was considered statistically significant. A statistical analysis was conducted using SPSS version 25.0, and significant differences were determined using Duncan’s test and one-way ANOVA (*p* < 0.05). Data processing was performed using Excel 2021. All results were analyzed using GraphPad Prism version 11.0, generating bar charts, line graphs, box-and-whisker plots, and correlation heatmaps. Heatmaps for the expression models of relevant genes in different maize tissues were generated using TB tools.

## 5. Conclusions

**Figure 12:**
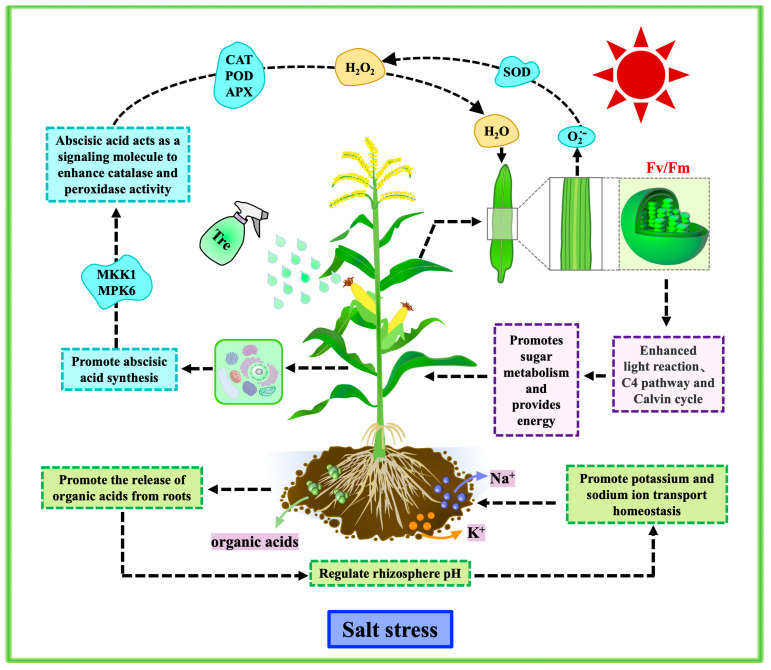
Mechanism diagram of trehalose alleviating salt stress in maize.

In summary, this study demonstrates that exogenous trehalose enhances salt tolerance in maize by regulating its growth, physiology, and gene expression under salt stress. We propose a simplified model to describe the role of exogenous trehalose in maize salt tolerance (Figure 12). First, the application of trehalose improves soil ecology, promoting maize growth under salt stress and partially restoring ion transport homeostasis. Furthermore, trehalose endows maize with stronger antioxidant enzyme activity, a lower accumulation of reactive oxygen species, and a higher relative water content, thereby enhancing its osmotic regulation capacity and oxidative stress resistance under salt stress. Additionally, maize treated with exogenous trehalose exhibits a higher chlorophyll content and stronger photosynthesis, resulting in a more positive effect on carbon dioxide fixation and nutritional growth, which mitigates the impact of salt stress on the production of energy substances and promotes biomass accumulation. Moreover, differentially expressed genes (DEGs) indicate that exogenous trehalose influences the signaling pathways of endogenous abscisic acid and the stress-responsive genes in the MAPK signaling pathway under salt stress, suggesting that trehalose may improve the adaptability of maize to adversity through complex plant hormone signaling networks and synthesis pathways. Understanding the mechanisms by which trehalose enhances maize tolerance will aid in the cultivation of crops adapted to salt stress and saline-alkali lands. However, further experiments are needed to uncover the specific molecular mechanisms underlying this effect, which may significantly contribute to improving crop yields.

## Data Availability

The data that support the findings of this study are available in the Supplementary Materials of this article.

## Supplementary Materials

The following supporting information can be downloaded at: https://www.mdpi.com/article/10.3390/plants13243506/s1, Table S1: Hydroponic maize seedling root morphology parameters; Table S2: Specific information on significant entries for GO and KEGG enrichment pathways; Table S3: Primer sequences used in this study; Figure S1: Analysis of differentially expressed genes (DEGs), Gene Ontology (GO), and Kyoto Encyclopedia of Genes and Genomes (KEGG) pathway enrichment under salt stress and trehalose treatment in maize; Figure S2: Trehalose and sugar metabolism transcription profiles in maize leaves under salt stress and trehalose treatment; Figure S3: Transcriptional differences in abscisic acid signaling and MAPK signaling pathways in maize leaves under salt stress and trehalose treatment; Table S4: The physiological parameters in the hydroponic experiment; Table S5: The indicator data of plants and soil in the soil cultivation experiment; Table S6: Heatmap data of transcriptomic metabolic pathways related to the experiment; Table S7: Relative expression levels of key genes under different treatments.
