# Supplementary material for: Combined Physiological and Transcriptomic Analyses of the Effects of Exogenous Trehalose on Salt Tolerance in Maize (Zea mays L.)
Source: Plants (Basel). 2024 Dec 16;13(24):3506. doi: 10.3390/plants13243506 (PMC11676066; doi:10.3390/plants13243506)
Supplement: Supplementary file 1 [file plants-13-03506-s001.zip › Figures S1-S3.pdf]

Supplementary Material

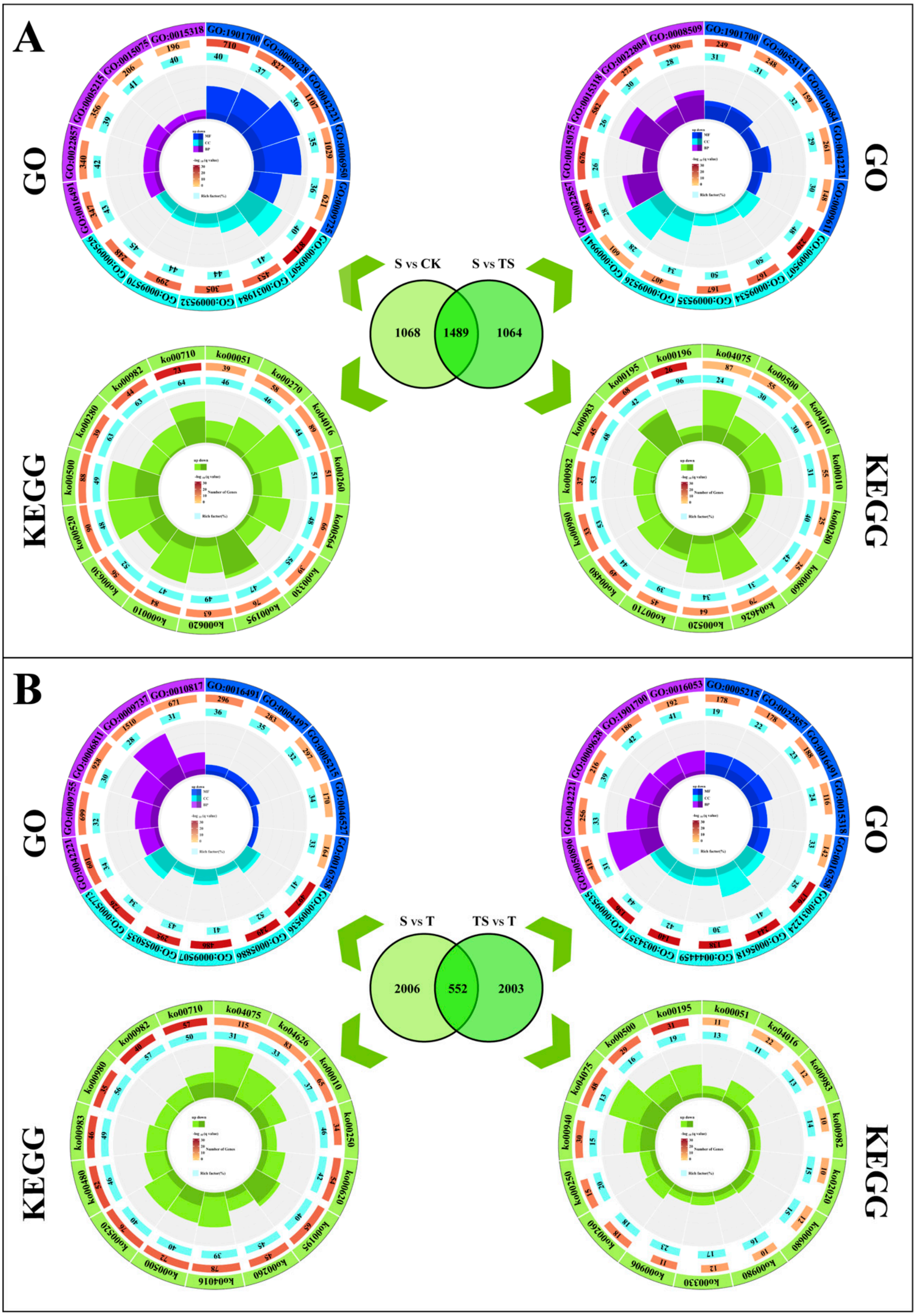

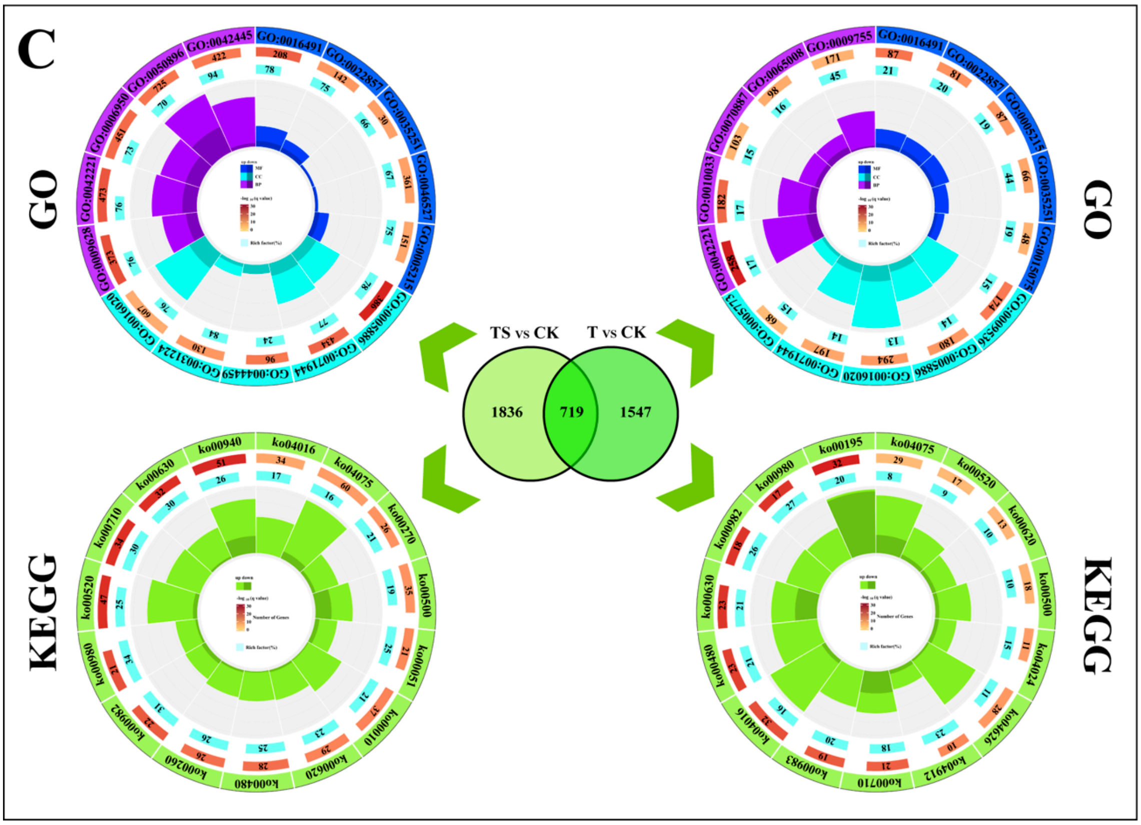

Figure S1. Analysis of differentially expressed genes (DEGs), Gene Ontology (GO), and Kyoto Encyclopedia of Genes and Genomes (KEGG) pathway enrichment under salt stress and trehalose treatment in maize. (A, B, C, D) Comparative analysis of DEGs, GO, and KEGG pathway enrichment under salt stress and trehalose treatment. Blue, green, and purple represent molecular function (MF), cellular component (CC), and biological process (BP), respectively. The first circle represents the top 15 GO terms or KEGG pathways. The second circle indicates significant entries for gene enrichment in the specified GO terms or KEGG pathways. The third circle shows the enrichment factor. The fourth circle indicates the number of genes for each GO term or KEGG pathway, with dark colors representing downregulated genes and light colors representing upregulated genes (*q* value < 0.05). GO entry information is detailed in supplementary Table S2.

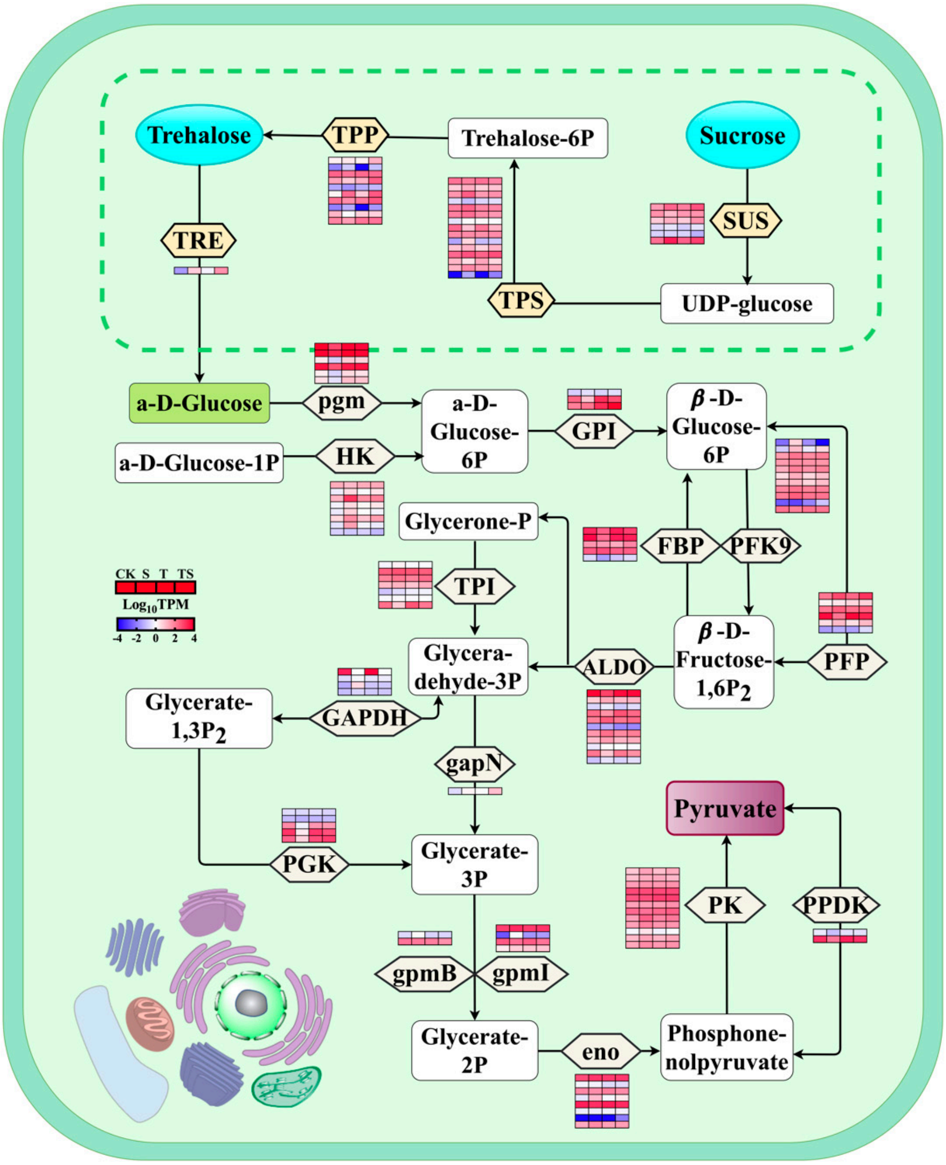

Figure S2. Trehalose and sugar metabolism transcription profiles in maize leaves under salt stress and trehalose treatment. The colored blocks represent log2 TPM values (CK: the control, S: salt stress, T: the application of trehalose under normal conditions, TS: the application of trehalose under salt stress). Red and blue indicate significantly upregulated and downregulated genes, respectively (log2 |TPM| ≥ 1, *q* value < 0.05, n=3). For enzyme reactions, arrows between two metabolites indicate the direction of the data decomposition reaction. Transport proteins and transcription factors are represented by yellow boxes, while metabolites are represented by white boxes. Key products related to trehalose metabolism are marked with blue squares. Green labels indicate metabolites connecting trehalose metabolism and sugar metabolism. Purple labels represent the final products of sugar metabolism. SUS: sucrose synthase; TPS: trehalose 6-phosphate synthase; TPP: trehalose 6-phosphate phosphatase; TRE: alpha, alpha-trehalase; HK: hexokinase; PGM: phosphoglucomutase; GPI: glucose-6-phosphate isomerase; FBPP: fructose-1,6-bisphosphatase I; PFK9: 6-phosphofructokinase; PFP: diphosphate-dependent phosphofructokinase; TPI: triosephosphate isomerase; ALDO: fructose-bisphosphate aldolase, class I; GAPHD: glyceraldehyde 3-phosphate dehydrogenase; PGK: phosphoglycerate kinase; PK: pyruvate kinase; PPDK: pyruvate, orthophosphate dikinase; gapN: glyceraldehyde-3-phosphate dehydrogenase; gpmB: 2,3-bisphosphoglycerate-dependent phosphoglycerate mutase; gpmI: 2,3-bisphosphoglycerate-independent phosphoglycerate mutase; eno: enolase 1/2/3.

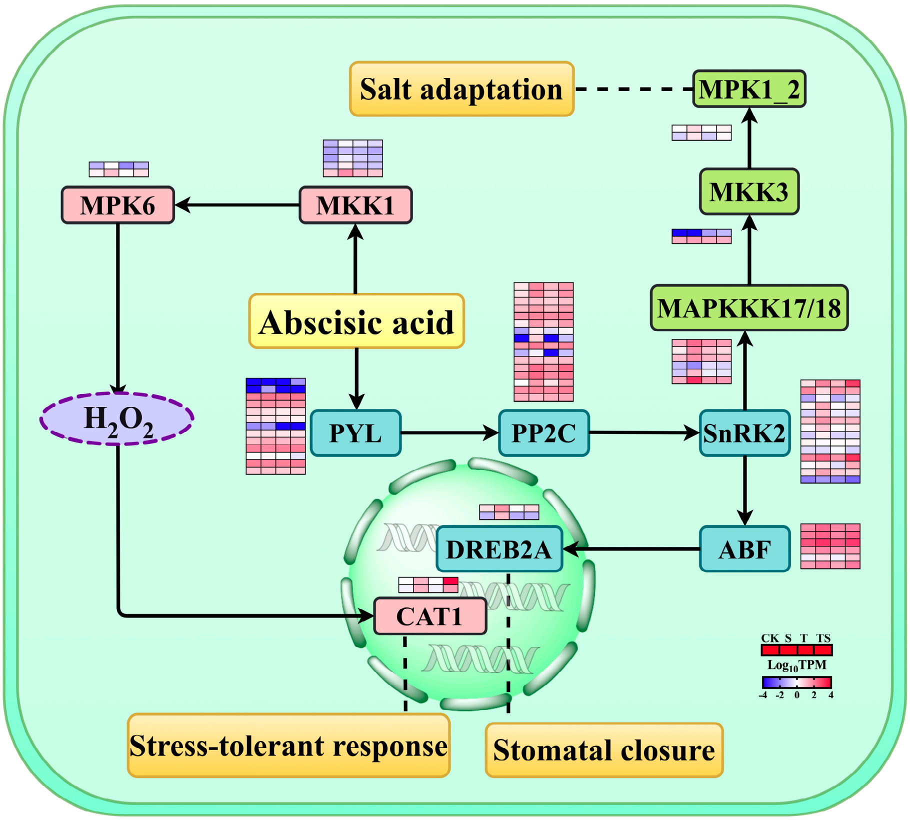

Figure S3. Transcriptional differences in abscisic acid signaling and MAPK signaling pathways in maize leaves under salt stress and trehalose treatment. The colored blocks represent log2 TPM values (CK: the control, S: salt stress, T: the application of trehalose under normal conditions, TS: the application of trehalose under salt stress). Red and blue indicate significantly upregulated and downregulated genes, respectively (log2 |TPM| ≥ 1, q value < 0.05, n=3). For enzyme reactions, the direction of arrows indicates the order of signal transduction. Salt adaptation signals are represented by green boxes, tolerance response signaling molecules by pink boxes, and stomatal closure signaling molecules by blue boxes. MPK6: mitogen-activated protein kinase 6; MKK1: mitogen-activated protein kinase kinase 1; CAT1: catalase; PYL: abscisic acid receptor PYR/PYL family; PP2C: protein phosphatase 2C; ABF: ABA responsive element binding factor; SnRK2: serine/threonine-protein kinase SRK2; MAPKKK17/18: mitogen-activated protein kinase kinase kinase 17/18; MKK3: mitogen-activated protein kinase kinase 3; MPK1_2: mitogen-activated protein kinase 1/2.
