# Supplementary material for: Combined Physiological and Transcriptomic Analyses of the Effects of Exogenous Trehalose on Salt Tolerance in Maize (Zea mays L.)
Source: Plants (Basel). 2024 Dec 16;13(24):3506. doi: 10.3390/plants13243506 (PMC11676066; doi:10.3390/plants13243506)
Supplement: Supplementary file 1 [file plants-13-03506-s001.zip › Table S1-S3.pdf]

Supplementary Material

Table S1. Hydroponic maize seedling root morphology parameters.

Treatment	time	Root length(cm)	Specific root length (m/g)	Average root diameter (mm)	Root surface area (cm^2)	Root volume (cm^3)
CK	0d	0.84±0.11ns	8.82±0.77ns	0.29±0.01ns	107.52±7.02ns	0.95±0.09ns
S		0.83±0.07	8.94±1.14	0.28±0.01	99.04±9.77	0.92±0.14
10T		0.82±0.05	8.43±1.20	0.29±0.01	96.13±8.90	0.88±0.03
10TS		0.83±0.03	9.11±1.45	0.29±0.01	95.39±10.76	0.89±0.05
CK	S1d	1.11±0.09b	12.7±1.38ab	0.35±0.01bc	146.44±5.34ab	1.17±0.04ab
S		0.97±0.11b	11.14±1.18b	0.32±0.01c	127.15±1.35c	1.11±0.03c
10T		1.68±0.19a	17.03±2.42a	0.36±0.01a	165.50±3.98a	1.53±0.07a
10TS		1.27±0.16ab	12.2±1.20ab	0.34±0.01b	153.56±4.47b	1.29±0.05bc
CK	S3d	1.58±0.19c	15.44±1.78b	0.38±0.01bc	180.18±4.80b	1.55±0.08ab
S		1.16±0.07b	12.83±0.11b	0.34±0.01c	151.26±2.96c	1.32±0.03c
10T		2.35±0.19a	21.44±1.26a	0.40±0.01ab	217.41±11.86a	1.72±0.03a
10TS		1.62±0.13b	14.37±1.50b	0.37±0.01a	174.11±4.46b	1.71±0.06b
CK	S7d	2.15±0.13b	20.01±2.22ab	0.40±0.01b	231.77±7.86b	2.03±0.16b
S		1.36±0.16c	13.71±1.30b	0.36±0.01c	178.89±5.46c	1.54±0.03c
10T		2.85±0.16a	27.74±2.64a	0.44±0.01a	301.42±10.29a	2.67±0.11a
10TS		1.93±0.10b	18.92±1.55ab	0.39±0.01b	214.8±16.06b	1.92±0.07b

[1] Lowercase letters indicate significant differences ($P < 0.05$) between groups under salt stress and trehalose treatment.

Table S2. Specific information on significant entries for GO and KEGG enrichment pathways.

Group	GO.ID	Term	totalnumber	termnumber	pvalue
TS-S	GO:0022857	transmembrane transporter activity	2161	425	1.18E-11
	GO:0005215	transporter activity	2161	446	3.49E-10
	GO:0015075	ion transmembrane transporter activity	2161	264	2.52E-10
	GO:0016491	oxidoreductase activity	2161	402	1.34E-17
	GO:0015318	inorganic molecular entity transmembrane transporter activity	2161	243	1.41E-06
	GO:0009507	chloroplast	2161	795	4.00E-11
	GO:0044436	thylakoid part	2161	510	8.81E-13
	GO:0009534	chloroplast thylakoid	2161	220	3.69E-22
	GO:0031976	plastid thylakoid	2161	222	5.47E-24
	GO:0009579	thylakoid	2161	265	9.60E-27
	GO:0009605	response to external stimulus	2161	677	0.01014
	GO:0006952	defense response	2161	470	0.00553091
	GO:0009628	response to abiotic stimulus	2161	945	0.00325929
	GO:0055114	oxidation-reduction process	2161	477	0.00019435
	GO:1901700	response to oxygen-containing compound	2161	852	2.66E-09
S-CK	GO:0016491	oxidoreductase activity	2161	456	8.87E-11
	GO:0022857	transmembrane transporter activity	2161	422	2.66E-09
	GO:0005215	transporter activity	2161	441	1.39E-05
	GO:0015075	ion transmembrane transporter activity	2161	261	0.00019435
	GO:0015318	inorganic molecular entity transmembrane transporter activity	2161	249	0.00325929
	GO:0009507	chloroplast	2161	1384	9.60E-27
	GO:0031984	organelle subcompartment	2161	651	1.81E-14
	GO:0009532	plastid stroma	2161	518	9.98E-14
	GO:0009570	chloroplast stroma	2161	506	4.25E-13
	GO:0009526	plastid envelope	2161	406	8.81E-13
	GO:1901700	response to oxygen-containing compound	2161	845	1.34E-17
	GO:0009628	response to abiotic stimulus	2161	1065	3.15E-11
	GO:0042221	response to chemical	2161	1355	3.49E-10
	GO:0006950	response to stress	2161	1268	1.61E-06
	GO:0009725	response to hormone	2161	765	0.00010056
T-TS	GO:0005215	transporter activity	2161	258	3.9293E-08
	GO:0022857	transmembrane transporter activity	2161	258	3.9293E-08
	GO:0016491	oxidoreductase activity	2161	270	3.211E-07
	GO:0015318	inorganic molecular entity transmembrane transporter activity	2161	166	1.2168E-05
	GO:0016758	transferase activity, transferring hexosyl groups	2161	198	0.00045067
	GO:0031224	intrinsic component of membrane	2161	280	1.44E-28
	GO:0005618	cell wall	2161	304	1.44E-28
	GO:0044459	plasma membrane part	2161	206	1.44E-28
	GO:0034357	photosynthetic membrane	2161	244	1.44E-28
	GO:0009535	plastid thylakoid membrane	2161	232	1.44E-28
	GO:0050896	response to stimulus	2161	568	6.6108E-11
	GO:0042221	response to chemical	2161	343	1.8888E-09
	GO:0009628	response to abiotic stimulus	2161	284	3.4103E-09
	GO:1901700	response to oxygen-containing compound	2161	241	3.4103E-08
	GO:0016053	organic acid biosynthetic process	2161	222	3.3332E-06

Table S2. Specific information on significant entries for GO and KEGG enrichment pathways.

Group	GO.ID	Term	totalnumber	termnumber	pvalue
T-CK	**GO:0005773**	vacuole	2161	**83**	**0.388608**
	**GO:0070887**	cellular response to chemical stimulus	2161	**126**	**0.17765941**
	**GO:0009755**	hormone-mediated signaling pathway	2161	**179**	**0.13624409**
	**GO:0015075**	ion transmembrane transporter activity	2161	**62**	**0.13050556**
	**GO:0065008**	regulation of biological quality	2161	**129**	**0.07032766**
	**GO:0071944**	cell periphery	2161	**238**	**0.055296**
	**GO:0035251**	UDP-glucosyltransferase activity	2161	**70**	**0.03917727**
	**GO:0016020**	membrane	2161	**368**	**0.012672**
	**GO:0005215**	transporter activity	2161	**105**	**0.01077375**
	**GO:0005886**	plasma membrane	2161	**216**	**0.010752**
	**GO:0022857**	transmembrane transporter activity	2161	**99**	**0.0051545**
	**GO:0009536**	plastid	2161	**178**	**0.00052992**
	**GO:0016491**	oxidoreductase activity	2161	**107**	**0.00027885**
	**GO:0010033**	response to organic substance	2161	**217**	**8.59E-05**
	**GO:0042221**	response to chemical	2161	**308**	**7.87E-08**
T-S	**GO:0016491**	oxidoreductase activity	2161	**409**	**2.43E-10**
	**GO:0004497**	monooxygenase activity	2161	**363**	**1.69E-07**
	**GO:0005215**	transporter activity	2161	**381**	**0.0001077**
	**GO:0046527**	glucosyltransferase activity	2161	**225**	**0.0018667**
	**GO:0016758**	transferase activity, transferring hexosyl groups	2161	**218**	**0.0045947**
	**GO:0009536**	plastid	2161	**875**	**1.27E-28**
	**GO:0005886**	plasma membrane	2161	**455**	**1.27E-28**
	**GO:0009507**	chloroplast	2161	**856**	**1.27E-28**
	**GO:0055035**	chloroplast thylakoid membrane	2161	**520**	**2.93E-25**
	**GO:0005773**	vacuole	2161	**1223**	**5.76E-25**
	**GO:0042221**	response to chemical	2161	**726**	**7.08E-16**
	**GO:0009755**	hormone-mediated signaling pathway	2161	**928**	**7.61E-11**
	**GO:0006811**	ion transport	2161	**1172**	**2.85E-09**
	**GO:0009737**	response to abscisic acid	2161	**1983**	**7.48E-08**
	**GO:0010817**	regulation of hormone levels	2161	**837**	**1.90E-07**
TS-CK	**GO:0016491**	oxidoreductase activity	2161	**208**	**1.0901E-05**
	**GO:0022857**	transmembrane transporter activity	2161	**142**	**0.00697125**
	**GO:0035251**	UDP-glucosyltransferase activity	2161	**30**	**0.0164775**
	**GO:0046527**	glucosyltransferase activity	2161	**36**	**0.0164775**
	**GO:0005215**	transporter activity	2161	**151**	**2.54E-02**
	**GO:0005886**	plasma membrane	2161	**386**	**2.30E-08**
	**GO:0071944**	cell periphery	2161	**434**	**3.63E-08**
	**GO:0044459**	plasma membrane part	2161	**96**	**8.352E-07**
	**GO:0031224**	intrinsic component of membrane	2161	**130**	**4.8384E-06**
	**GO:0016020**	membrane	2161	**607**	**2.4686E-05**
	**GO:0009628**	response to abiotic stimulus	2161	**373**	**8.34E-10**
	**GO:0042221**	response to chemical	2161	**473**	**1.67E-06**
	**GO:0006950**	response to stress	2161	**451**	**4.09E-06**
	**GO:0050896**	response to stimulus	2161	**725**	**3.2195E-05**
	**GO:0042445**	hormone metabolic process	2161	**422**	**0.00012713**

Table S2. Specific information on significant entries for GO and KEGG enrichment pathways.

Group	KEGG.ID	Term	totalnumber	termnumber	pvalue
TS-S	ko04075	Plant hormone signal transduction	637	114	0.151128531
	ko00500	Starch and sucrose metabolism	637	78	0.004286
	ko04016	MAPK signaling pathway - plant	637	68	0.003117315
	ko00010	Glycolysis / Gluconeogenesis	637	81	0.003117315
	ko00280	Valine, leucine and isoleucine degradation	637	28	0.002292056
	ko00860	Porphyrin metabolism	637	43	0.001481136
	ko04626	Plant-pathogen interaction	637	90	0.000228913
	ko00520	Amino sugar and nucleotide sugar metabolism	637	78	7.50E-05
	ko00710	Carbon fixation in photosynthetic organisms	637	79	2.89E-05
	ko00480	Glutathione metabolism	637	54	2.16E-07
	ko00980	Metabolism of xenobiotics by cytochrome P450	637	35	1.95E-07
	ko00982	Drug metabolism - cytochrome P450	637	41	3.56E-08
	ko00983	Drug metabolism - other enzymes	637	47	3.00E-08
	ko00195	Photosynthesis	637	130	5.60E-09
	ko00196	Photosynthesis - antenna proteins	637	40	2.50E-15
S-CK	ko00051	Fructose and mannose metabolism	637	48	0.016948453
	ko00270	Cysteine and methionine metabolism	637	68	0.003593845
	ko04016	MAPK signaling pathway - plant	637	96	0.001646176
	ko00260	Glycine, serine and threonine metabolism	637	66	0.000598238
	ko00564	Glycerophospholipid metabolism	637	77	0.000572851
	ko00330	Arginine and proline metabolism	637	46	0.000572851
	ko00195	Photosynthesis	637	146	0.000554729
	ko00620	Pyruvate metabolism	637	78	0.000436407
	ko00010	Glycolysis / Gluconeogenesis	637	107	0.000190952
	ko00630	Glyoxylate and dicarboxylate metabolism	637	87	0.00013818
	ko00520	Amino sugar and nucleotide sugar metabolism	637	108	6.13E-05
	ko00500	Starch and sucrose metabolism	637	129	3.86E-05
	ko00280	Valine, leucine and isoleucine degradation	637	43	1.37E-05
	ko00982	Drug metabolism - cytochrome P450	637	48	4.19E-06
	ko00710	Carbon fixation in photosynthetic organisms	637	119	2.50E-10
T-TS	ko00051	Fructose and mannose metabolism	637	15	0.347420775
	ko04016	MAPK signaling pathway - plant	637	28	0.34395832
	ko00983	Drug metabolism - other enzymes	637	18	0.342507133
	ko00982	Drug metabolism - cytochrome P450	637	15	0.333398889
	ko02020	Two-component system	637	13	0.253423011
	ko00680	Methane metabolism	637	20	0.206099091
	ko00980	Metabolism of xenobiotics by cytochrome P450	637	15	0.206099091
	ko00330	Arginine and proline metabolism	637	16	0.102675575
	ko00906	Carotenoid biosynthesis	637	16	0.015951084
	ko00260	Glycine, serine and threonine metabolism	637	23	0.012509501
	ko00250	Alanine, aspartate and glutamate metabolism	637	20	0.012355432
	ko00940	Phenylpropanoid biosynthesis	637	44	0.007860585
	ko04075	Plant hormone signal transduction	637	66	0.005163185
	ko00500	Starch and sucrose metabolism	637	40	0.005163185
	ko00195	Photosynthesis	637	35	0.000147836

Table S2. Specific information on significant entries for GO and KEGG enrichment pathways.

Group	KEGG.ID	Term	totalnumber	termnumber	pvalue
T-CK	ko04075	Plant hormone signal transduction	637	38	0.489517783
	ko00520	Amino sugar and nucleotide sugar metabolism	637	19	0.375742346
	ko00620	Pyruvate metabolism	637	15	0.295771379
	ko00500	Starch and sucrose metabolism	637	20	0.215771237
	ko04024	cAMP signaling pathway	637	13	0.055767686
	ko04626	Plant-pathogen interaction	637	29	0.037321403
	ko04912	GnRH signaling pathway	637	12	0.008612434
	ko00710	Carbon fixation in photosynthetic organisms	637	32	0.00047602
	ko00983	Drug metabolism - other enzymes	637	19	0.000290847
	ko04016	MAPK signaling pathway - plant	637	34	0.000139235
	ko00480	Glutathione metabolism	637	23	4.4011E-05
	ko00630	Glyoxylate and dicarboxylate metabolism	637	34	2.55976E-05
	ko00982	Drug metabolism - cytochrome P450	637	18	2.55976E-05
	ko00980	Metabolism of xenobiotics by cytochrome P450	637	17	2.55976E-05
	ko00195	Photosynthesis	637	63	4.65279E-06
T-S	ko04075	Plant hormone signal transduction	637	148	0.009813243
	ko04626	Plant-pathogen interaction	637	95	0.009813243
	ko00010	Glycolysis / Gluconeogenesis	637	89	0.001715694
	ko00250	Alanine, aspartate and glutamate metabolism	637	46	0.000452114
	ko00620	Pyruvate metabolism	637	69	8.53E-05
	ko00195	Photosynthesis	637	121	8.31E-05
	ko00260	Glycine, serine and threonine metabolism	637	62	8.31E-05
	ko04016	MAPK signaling pathway - plant	637	88	7.58E-05
	ko00500	Starch and sucrose metabolism	637	105	6.11E-05
	ko00520	Amino sugar and nucleotide sugar metabolism	637	93	1.91E-05
	ko00480	Glutathione metabolism	637	58	8.83E-06
	ko00983	Drug metabolism - other enzymes	637	49	4.94E-06
	ko00980	Metabolism of xenobiotics by cytochrome P450	637	37	2.86E-06
	ko00982	Drug metabolism - cytochrome P450	637	44	2.49E-07
	ko00710	Carbon fixation in photosynthetic organisms	637	91	1.84E-07
TS-CK	ko04016	MAPK signaling pathway - plant	637	34	0.099554292
	ko04075	Plant hormone signal transduction	637	67	0.040630802
	ko00270	Cysteine and methionine metabolism	637	29	0.024763433
	ko00500	Starch and sucrose metabolism	637	50	0.024204624
	ko00051	Fructose and mannose metabolism	637	25	0.008414916
	ko00010	Glycolysis / Gluconeogenesis	637	38	0.005635472
	ko00620	Pyruvate metabolism	637	31	0.005635472
	ko00480	Glutathione metabolism	637	29	0.001609265
	ko00260	Glycine, serine and threonine metabolism	637	28	0.001585022
	ko00982	Drug metabolism - cytochrome P450	637	26	0.000302264
	ko00980	Metabolism of xenobiotics by cytochrome P450	637	23	0.000148765
	ko00520	Amino sugar and nucleotide sugar metabolism	637	51	1.47E-05
	ko00710	Carbon fixation in photosynthetic organisms	637	34	1.47E-05
	ko00630	Glyoxylate and dicarboxylate metabolism	637	34	1.47E-05
	ko00940	Phenylpropanoid biosynthesis	637	67	9.40E-06

Table S3. Primer sequences used in this study.

Primer Name	Forward Primer (5′-3′)	Reverse Primer (3′-5′)
ZmTRE1	CCGTCACCGTATCCTCAGAT	AATGTTACACTGCTCGTCGG
ZmTPP2	AATGTGGCGGAAGATGACTAT	GGCAAGGGTAACCTTCCAAA
ZmSNRK2.12	GGCACCCTAACATCATCCAG	AAATAGTTCACCACCAGCCG
ZmPP2C6	CCAAGCTAATTTCTCCCCCG	AAAGCGTCTGGTTGGTAACT
ZmPYL9	ACAAAAACCGACTAGCCCAG	TGAGGACGAGACAAAGGAGT
EF-1a	CCGTCACCGTATCCTCAGAT	AATGTTACACTGCTCGTCGG
